# Green Superhydrophobic Surfaces: From Natural Substrates to Sustainable Fabrication Processes

**DOI:** 10.3390/ma18184270

**Published:** 2025-09-12

**Authors:** Siyuan Wang, Hengyuan Liu, Gang Liu, Pengfei Song, Jingyi Liu, Zhao Liang, Ding Chen, Guanlin Ren

**Affiliations:** 1 Engineering Research Center of Additive Manufacturing Aeronautical Materials of Henan Province, Nanyang Institute of Technology, Nanyang 473004, China; 2 School of New Materials Engineering, Zhengzhou Technical College, Zhengzhou 450010, China; 3 State Key Laboratory of Advanced Design and Manufacturing for Vehicle Body, Hunan University, Changsha 410082, China; 4 Institute of Micro/Nano Materials and Devices, Ningbo University of Technology, Ningbo 315211, China; 5 State Key Laboratory of Solid Lubrication, Lanzhou Institute of Chemical Physics, Chinese Academy of Sciences, Lanzhou 730000, China

**Keywords:** superhydrophobic coatings, green process, bio-based materials, surface engineering, multifunctional applications

## Abstract

Superhydrophobic surfaces, characterized by water contact angles greater than 150°, have attracted widespread interest due to their exceptional water repellency and multifunctional applications. However, traditional fabrication methods often rely on fluorinated compounds and petroleum-based polymers, raising environmental and health concerns. In response to growing environmental and health problems, recent research has increasingly focused on developing green superhydrophobic surfaces, employing eco-friendly materials, energy-efficient processes, and non-toxic modifiers. This review systematically summarizes recent progress in the development of green superhydrophobic materials, focusing on the use of natural substrates such as cellulose, chitosan, starch, lignin, and silk fibroin. Sustainable fabrication techniques, including spray coating, dip coating, sol–gel processing, electrospinning, laser texturing, and self-assembly, are critically discussed with regards to their environmental compatibility, scalability, and integration with biodegradable components. Furthermore, the functional performance of these coatings is explored in diverse application fields, including self-cleaning, oil–water separation, anti-corrosion, anti-icing, food packaging, and biomedical devices. Key challenges such as mechanical durability, substrate adhesion, and large-scale processing are addressed, alongside emerging strategies that combine green chemistry with surface engineering. This review provides a comprehensive perspective on the design and deployment of eco-friendly superhydrophobic surfaces, aiming to accelerate their practical implementation across sustainable technologies.

## 1. Introduction

Superhydrophobic surfaces, characterized by water contact angles (WCAs) exceeding 150° and low sliding angles (SAs), exhibit exceptional water repellency by mimicking natural phenomena such as the self-cleaning “lotus effect” observed in lotus leaves and insect cuticles [1]. These surfaces have garnered significant scientific and technological interest due to their potential in enabling a wide array of applications, including self-cleaning materials [2,3], antifouling coatings [4,5], drag reduction [6,7], anti-icing systems [8,9], metal surface protection [10,11], and oil–water separation [12,13]. A unique combination of surface roughness and low surface energy underpins their remarkable wettability control, making them a focal point in functional interface engineering.

However, conventional methods for fabricating superhydrophobic coatings often rely on fluorinated compounds and petroleum-based polymers, which raise serious environmental and health concerns [14,15,16]. These materials are not only energy-intensive to produce but also exhibit poor degradability, contributing to persistent organic pollution and bioaccumulation in ecosystems [17,18,19]. In response to the increasing demand for sustainable solutions, research in recent decades has shifted toward green chemistry approaches, aiming to replace synthetic or hazardous substances with naturally derived and biodegradable alternatives. Renewable biopolymers, such as cellulose, chitosan, lignin, starch, and silk fibroin, alongside mineral-based fillers, have emerged as promising candidates for constructing eco-friendly superhydrophobic systems [20,21,22,23].

**Figure 1:**
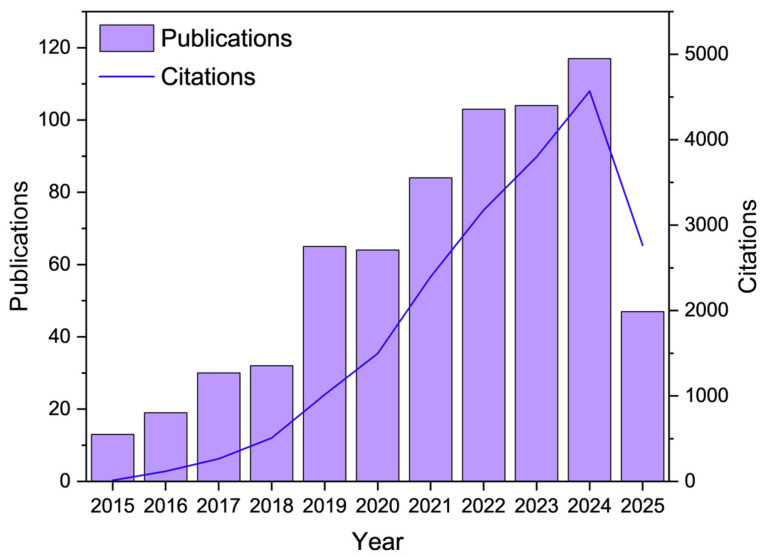
Number of times cited and publications, 2015–2025. The data were compiled from the Web of Science database on 28 July 2025. (The topic words for retrieval are “superhydrophobic” and “green materials”).

Recent bibliometric analysis further illustrates the growing academic and industrial interest in this field. A literature search using the Web of Science Core Collection, with the combined topic keywords “superhydrophobicity” and “green materials,” revealed a significant increase in publications and citations from 2015 to 2025 (Figure 1). This upward trend reflects the expanding research focus on environmentally sustainable alternatives to conventional superhydrophobic technologies. The intensification of research during this period underscores the need to systematically consolidate emerging knowledge across natural raw materials, eco-friendly processing techniques, and multifunctional applications.

Moreover, fabrication technologies have evolved to accommodate these green materials through more sustainable processing routes. Techniques such as spray coating, dip coating, sol–gel synthesis, electrospinning, laser ablation, and self-assembly are being reengineered to minimize toxic solvent usage, reduce energy consumption, and enable large-area or scalable production [24,25,26,27,28,29,30]. These innovations support the transition from laboratory demonstrations to practical implementation in sectors ranging from environmental remediation to biomedical engineering. Despite these advances, the current literature lacks a unified and in-depth summary that systematically links renewable material sources with eco-friendly processing technologies and functional outcomes across diverse application domains. Most existing reviews tend to focus either on material chemistry or fabrication techniques in isolation, without sufficiently addressing their interdependence or evaluating their environmental impacts. To address this gap, this review offers a holistic synthesis of recent advancements in sustainable superhydrophobic materials, uniquely emphasizing the synergistic interplay between natural substrates, green fabrication methods, and application-driven performance.

This review aims to comprehensively synthesize recent advancements in environmentally benign superhydrophobic coatings, with a specific emphasis on natural substrates and sustainable fabrication techniques. It highlights the functional versatility of these materials across various application domains, including self-cleaning, anti-corrosion, anti-icing, oil–water separation, and biomedical interfaces. While aspects such as mechanical robustness and weathering resistance are not discussed in depth, these remain important future directions for achieving real-world viability. The discussion further explores the key technical challenges that impact coatings, while also outlining emerging trends in linking material science with green engineering principles. By articulating the scientific foundation, technological significance, and real-world applicability of green superhydrophobic coatings, this work seeks to guide future research and to promote their transition from laboratory prototypes to industrial reality.

To provide a clear roadmap for readers, the structure of this review is outlined as follows: Section 2 introduces representative categories of green superhydrophobic materials, focusing on their renewable sources and eco-friendly materials; Section 3 presents key sustainable fabrication strategies, such as sol–gel processing, spray coating, and self-assembly; Section 4 highlights major application areas, including textiles, anti-corrosion surfaces, oil–water separation, food packaging, and biomedical devices.; Section 5 outlines current challenges, development trends, and techno-economic considerations that influence industrial feasibility; finally, Section 6 concludes the review with a summary of findings and future perspectives.

## 2. Natural Substrates for Superhydrophobic Surface

Increasing regulatory pressure on hazardous chemicals has driven the shift toward sustainable and biodegradable materials in surface coating technologies. Regulatory frameworks such as the European REACH and the United States EPA have already restricted the use of persistent organic pollutants, including long-chain perfluorinated compounds and certain reactive silanes [31,32]. In this context, natural polymers such as cellulose, chitosan, starch, and lignin provide not only functional benefits but also a path towards regulatory compliance. Their adoption supports both environmental safety and conformity with evolving international standards for chemical use and waste management.

Recent research has explored many renewable polymers and biopolymers as building blocks or binders for superhydrophobic coatings. These include cellulose, lignin, chitosan, starch, silk fibroin, and plant/animal waxes or proteins, among others. Such materials are typically abundant, low-cost, and often biodegradable. In coatings, they can form micro/nanostructures or serve as supports for nanoparticles, thereby reducing reliance on synthetic (especially fluorinated) chemicals.

### 2.1. Cellulose

Cellulose, the most abundant natural polymer on Earth, is a linear polysaccharide composed of *β*-1,4-linked glucopyranose units and is widely derived from renewable sources such as wood pulp, cotton, and nanostructured derivatives including cellulose nanocrystals (CNCs) and nanofibrillated cellulose (NFC). These nanocellulose forms, distinguished by their high aspect ratio, excellent mechanical properties, and large specific surface area, serve as structurally robust scaffolds for fabricating superhydrophobic coatings. Beyond their renewability and biodegradability, cellulose-based materials inherently facilitate the construction of the hierarchical surface roughness that is essential for achieving WCAs exceeding 150°.

The superhydrophobic performance of cellulose-based coatings originates not only from surface roughness but also from the intrinsic hydrogen bonding network within and between cellulose chains. Each cellulose monomer possesses multiple hydroxyl groups that engage in extensive intra- and intermolecular hydrogen bonding, which contributes to high crystallinity and structural rigidity. These interactions help cellulose maintain film integrity and resistance to deformation during processing or surface modification. Furthermore, hydroxyl groups serve as reactive sites for surface functionalization via esterification, silanization, or the grafting of hydrophobic moieties, allowing cellulose fibers to be transformed into water-repellent substrates [33]. When combined with inorganic nanoparticles or hierarchical structuring, these modified cellulose materials form stable, durable, and environmentally benign superhydrophobic surfaces. Recent studies have demonstrated diverse strategies for utilizing cellulose in superhydrophobic surface engineering. Huang et al. [34] developed a mechanically durable superhydrophobic coating by spraying an ethanol-based NFC suspension onto wood surfaces, followed by chemical vapor deposition (CVD) to reduce surface energy. The resulting coatings achieved a WCA of 161° and excellent abrasion resistance, exhibiting self-cleaning capabilities owing to the synergistic combination of nanocellulose-induced surface texture and post-modification chemistry. Similarly, Zhang et al. [35] employed α-cellulose 10-undecenoyl ester to fabricate superhydrophobic paper, achieving a WCA of 152° with advancing and receding angles of 165° and 144°, respectively. The modified material exhibited significant thermal resistance and chemical stability, demonstrating the potential of chemically modified cellulose for environmental applications.

**Figure 2:**
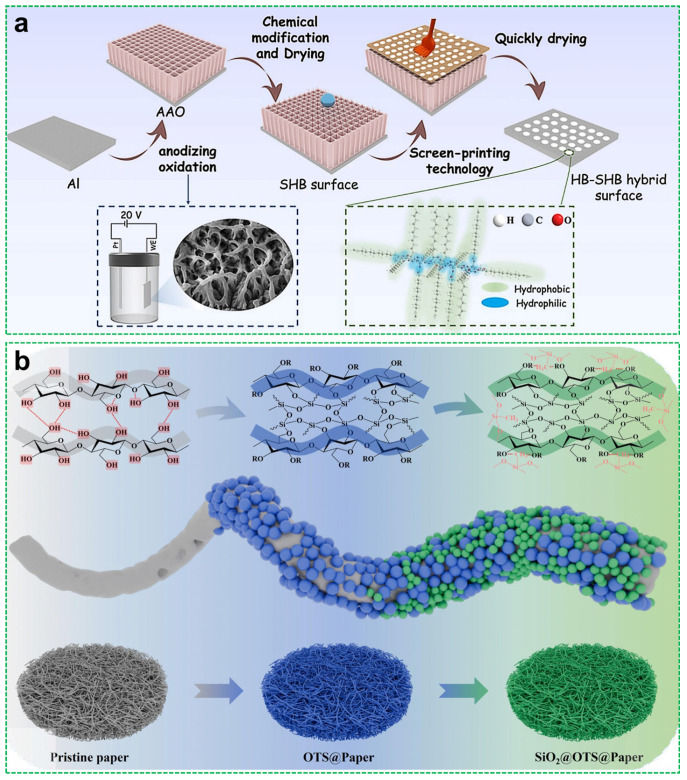
(**a**) Preparation Process of the HB–SHB Hybrid Surface. Reprinted with permission from [37]. Copyright © 2024 American Chemical Society. (**b**) Schematic illustration of the preparation of SiO_2_@OTS@Paper [38]. Copyright © 2025, Elsevier B.V.

Further extending its functionality, Chen et al. [36] introduced silanized cellulose into poly(L-lactic acid) via a solvent-evaporation-induced phase separation method, resulting in composite films with hierarchical micro/nano structures and tunable wettability. These films exhibited controlled water adhesion (with a maximum WCA of 160.2°), improved mechanical performance (e.g., elongation at breakup to 22.36%), and biodegradability, making them promising for antifouling and self-cleaning applications. Zhan et al. [37] adopted a wetting gradient strategy by partially acylating microcrystalline cellulose and depositing it onto anodized aluminum oxide (AAO), as shown in Figure 2a. The acylated cellulose created localized hydrophilic domains on an otherwise hydrophobic matrix, facilitating water droplet coalescence and enhancing fog harvesting efficiency to 1.5092 g/cm^2^/h, which is 1.77 times higher than that of unmodified surfaces.

In the domain of oil–water separation, cellulose-derived superhydrophobic materials have attracted significant attention. Ahmed S. Belal et al. [39] prepared a highly effective separation membrane by growing Cu(OH)_2_ nanorods on cellulose paper, followed by hydrophobic modification, achieving a contact angle of 169.7° and strong performance under harsh conditions. Ning et al. [38] fabricated a superhydrophobic cellulose paper through a sequential two-step immersion process, first in octadecyltrichlorosilane (OTS) to form polyorganosiloxane clusters, followed by the deposition of hydrophobic SiO_2_ nanoparticles (Figure 2b). This approach utilized the inherent porosity of cellulose and generated dual-scale surface structures during modification. The resultant material exhibited superhydrophobicity, with a WCA of 157°, and superoleophilicity. Consequently, it achieved a high separation efficiency of 99.31% for heavy oil–water mixtures, accompanied by fluxes exceeding over 5100 L/(m^2^·h).

Beyond individual case studies, Teisala et al. [40] provided a comprehensive review of superhydrophobic coatings based on cellulose substrates such as paper and cotton. They emphasized fabrication methods, including plasma treatment and CVD, and pointed out that the intrinsic fibrous morphology and porosity of cellulose facilitate the formation of the multiscale roughness patterns required for water repellency. These features, combined with the material’s compatibility with low-toxicity surface chemistries, enable simple yet scalable routes to superhydrophobicity.

In summary, cellulose offers a compelling combination of structural tunability, environmental compatibility, and abundant availability, making it a leading candidate in the development of green superhydrophobic materials. Its inherent microstructure and chemical modifiability support a broad spectrum of sustainable applications, ranging from self-cleaning surfaces and fog collection to oil–water separation and antifouling coatings.

### 2.2. Lignin

Lignin, a complex aromatic biopolymer accounting for approximately 15–30% of lignocellulosic biomass, plays a crucial structural role in plant cell walls by imparting rigidity, hydrophobicity, and resistance to microbial degradation. Chemically, lignin consists of highly cross-linked phenolic units primarily derived from monolignols coniferyl and sinapyl alcohols. As a major byproduct of the pulp and paper industry—particularly in the form of kraft lignin—it is produced in large quantities and is available at low cost, making it an attractive raw material for sustainable applications. Its intrinsic hydrophobicity, thermal stability, and abundance make lignin particularly suitable for developing environmentally benign superhydrophobic coatings.

**Figure 3:**
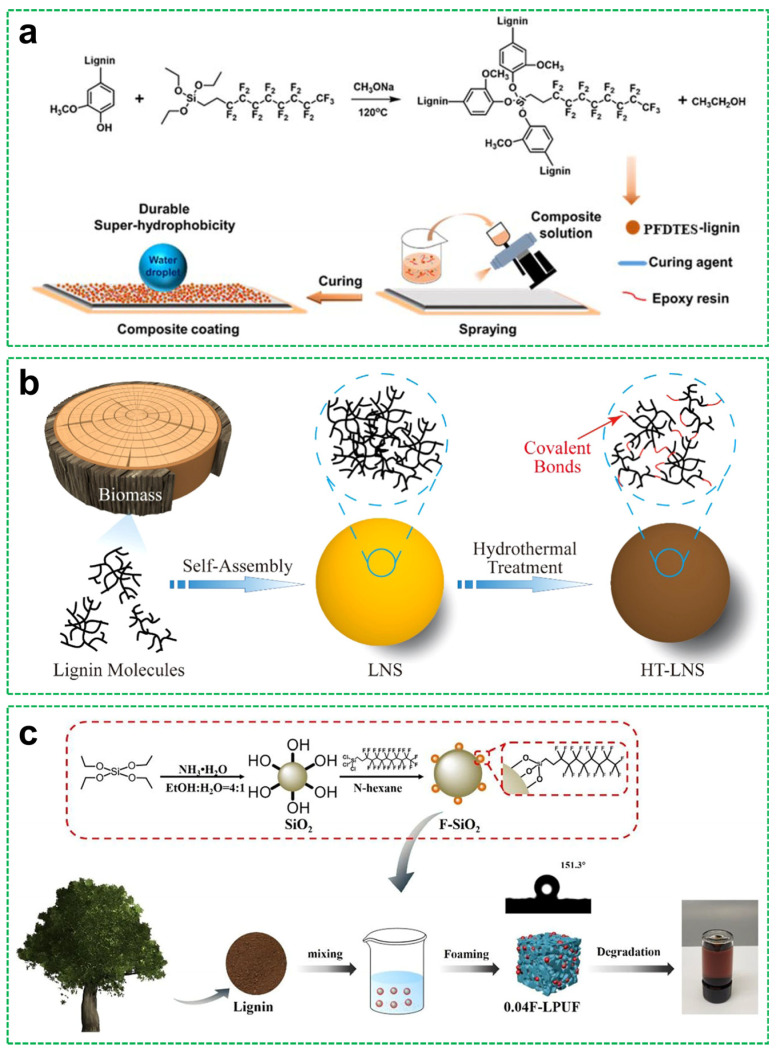
(**a**) Schematic illustration of lignin modification and the durable superhydrophobic coating fabrication process [41]. (**b**) Schematic diagram of the efficient preparation of heat-treated lignin nanospheres (HT-LNS). Reprinted with permission from [43]. Copyright © 2021 American Chemical Society. (**c**) Schematic representation of the preparation and degradation of superhydrophobic 0.04F-LPUF foam adsorbent [44]. Copyright © 2022 Elsevier B.V.

Recent studies have explored the functionalization of lignin to enhance its surface properties and its integration into hierarchical coating systems. Liu et al. [41] reported the synthesis of lignin-based superhydrophobic powders through the substitution reaction of kraft lignin with perfluorodecyltriethoxysilane (PFDTES), as shown in Figure 3a. The modified lignin, exhibiting both high surface roughness and low surface energy, was subsequently deposited onto various substrates via spray coating, resulting in durable superhydrophobic surfaces, achieving a WCA of 164.7°. Building upon the self-assembly potential of lignin, Ma et al. [42] fabricated lignin micro- and nanospheres with controllable particle sizes in a *γ*-valerolactone/water solvent system. A tailored morphology suitable for superhydrophobic wood coatings was achieved by modulating the reaction dynamics, wherein lignin acted as a green matrix for surface functionalization. Lignin provided structural support for micro/nano-roughness, resulting in a WCA of 164.4° and an SA of 5°.

Further advancing lignin nanostructures, Wang et al. [43] developed heat-treated lignin nanospheres (HT-LNS) stabilized by both covalent and non-covalent interactions. These nanospheres were obtained via hydrothermal treatment (Figure 3b), during which lignin molecules underwent condensation and cross-linking reactions, enabling the formation of structurally stable particles for use in superhydrophobic coatings, with the lignin enhancing solvent resistance and structural stability, achieving a WCA of 151.9° and an SA of 9.4°. Taking a different approach, Wu et al. [44] synthesized a lignin-based polyurethane (PU) foam via a one-step foaming process, incorporating F-SiO_2_ nanoparticles into a liquefied lignin polyol matrix, as shown in Figure 3c. This composite not only exhibited excellent oil adsorbing and superhydrophobic performance (with a WCA of 151.3°), but also demonstrated photothermal properties, highlighting the multifunctionality of lignin as a renewable component in advanced material design.

Collectively, these studies illustrate the versatility of lignin in enabling the fabrication of bio-based superhydrophobic materials. Through structural modification and nanoscale engineering, lignin can be effectively transformed into functional building blocks for durable, environmentally friendly coatings with potential applications in water repellency, oil–water separation, and thermal regulation.

### 2.3. Chitosan

Chitosan, a linear polysaccharide composed of glucosamine units, is obtained through the deacetylation of chitin, which is abundantly found in shellfish exoskeletons and fungal cell walls. Due to its biodegradability, biocompatibility, non-toxicity, and inherent antimicrobial activity, chitosan has attracted significant attention as a sustainable polymeric material. In the field of superhydrophobic coatings, chitosan functions effectively as a binder or matrix material. The hydroxyl and amino groups in chitosan are capable of forming extensive hydrogen bonding networks, both intra- and intermolecularly, contributing to structural cohesion. Additionally, the amino groups can participate in ionic interactions or covalent cross-linking reactions with aldehyde-containing compounds (e.g., glutaraldehyde), enhancing mechanical stability and water resistance. These interactions play a pivotal role in anchoring chitosan onto inorganic nanoparticles or rough substrates in superhydrophobic coatings. Furthermore, its excellent film-forming ability and widespread natural availability make chitosan a promising candidate for environmentally friendly surface engineering.

**Figure 4:**
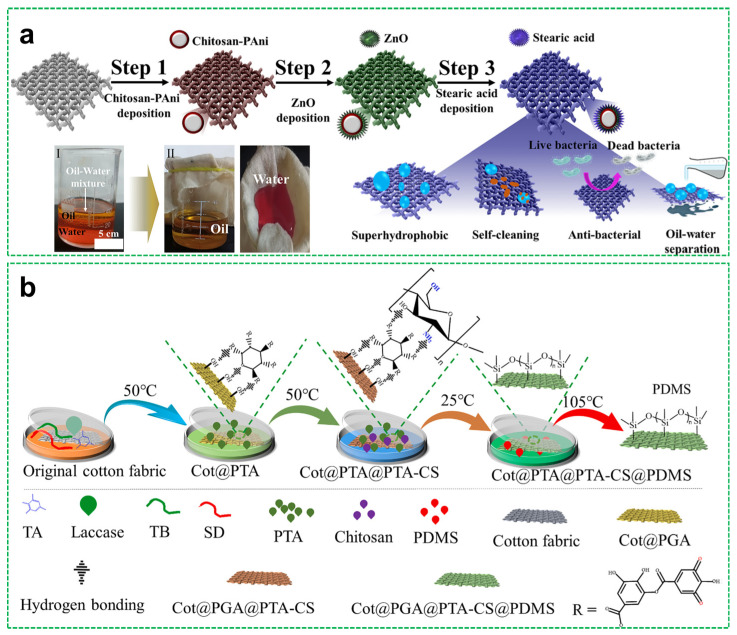
(**a**) Schematic representation of the fabrication of superhydrophobic cotton using chitosan– PAni–ZnO–STA composite coating [46]. Copyright © 2023 Elsevier B.V. (**b**) Illustration of the superhydrophobic, UV-resistant, anti-oxidative, and photothermal cotton fabrics [47]. Copyright © 2023 Elsevier B.V.

Several studies have demonstrated the utility of chitosan in constructing fluorine-free superhydrophobic systems. For example, chitosan has been combined with silica nanoparticles and polydimethylsiloxane (PDMS) to form spray-coated textiles that exhibit high water repellency. Raeisi et al. [45] employed a dip-coating method to fabricate chitosan/TiO_2_ nanocomposite coatings on cotton fabrics. In this system, chitosan served as a binder, enhancing nanoparticle adhesion and enabling the formation of a densely packed nanoscale architecture. The resulting surface demonstrated not only superhydrophobicity (achieving a WCA of 161°), but also antibacterial activity and ultraviolet protection. Similarly, Suryaprabha et al. [46] developed a multifunctional superhydrophobic coating using a chitosan–polyaniline–ZnO–stearic acid (Pani–ZnO–STA) composite. Chitosan acted as both a structural matrix and an antibacterial agent, facilitating the anchoring of ZnO nanoparticles and stearic acid. The synergistic assembly of these components produced micro/nano-scale surface roughness, achieving a WCA of 154.4°, enabling excellent water repellency and self-cleaning behavior, as shown in Figure 4a.

Opting for a fluorine-free approach, Zheng et al. [47] fabricated a photothermal and UV-resistant superhydrophobic cotton by coating it with a chitosan–tannic acid complex (PTA–CS), followed by PDMS treatment (Figure 4b). Chitosan formed a hydrophobic layer through hydrogen bonding interactions with tannic acid, while PDMS further enhanced surface roughness. The final coating exhibited excellent superhydrophobic (achieving a WCA of 153.3°), self-cleaning capabilities, and efficient oil–water separation, along with retained photothermal and UV-shielding properties derived from chitosan. Additionally, Tagliaro et al. [48] synthesized superhydrophobic coatings by modifying chitosan with stearic acid and applying solvent-free thermal treatment to textile substrates. The modified chitosan reduced surface energy and formed a uniform film with hierarchical roughness, resulting in durable, biocompatible superhydrophobic coatings with advancing/receding contact angles of 151° and 136°, respectively.

Overall, these studies underscore the versatility of chitosan as a green and multifunctional component in the design of superhydrophobic surfaces. Its intrinsic bioactivity, combined with its chemical reactivity and compatibility with various nanomaterials, enables the development of sustainable coatings with potential applications in textiles, environmental remediation, and biomedicine.

### 2.4. Starch

Starch is a naturally abundant and cost-effective polysaccharide derived from plant sources such as corn, potatoes, and rice. It is composed of two glucose-based macromolecules, both consisting of α-1,4 and α-1,6 glycosidic linkages: the linear amylose and the highly branched amylopectin. Although native starch is inherently hydrophilic due to the presence of abundant hydroxyl groups, its chemical structure allows for facile functionalization. Through esterification, hydrophobic modification, or incorporation with waxes and other low-surface-energy substances, starch can be transformed into a viable candidate for superhydrophobic coatings. Additionally, starch granules ranging from 0.1 to 200 μm in size can undergo gelatinization and subsequent film formation, offering a flexible platform for surface engineering. While pure starch readily absorbs water, its combination with hydrophobic agents, such as lignin derivatives or fatty acid esters, can significantly improve water repellency.

**Figure 5:**
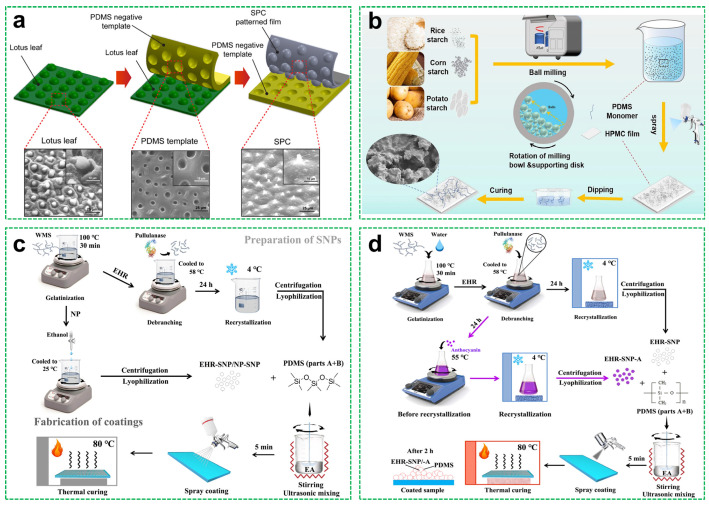
(**a**) Schematic illustrations of the lotus-inspired multistep process for fabricating patterned SPC surfaces. Reprinted with permission from [49]. Copyright © 2021 American Chemical Society. (**b**) Schematic illustration of the fabrication of HPMC films with superhydrophobic surfaces [50]. Copyright © 2024 Elsevier B.V. (**c**) The schematic diagram for the preparation of starch nanoparticles (SNPs) and nano-starch-based superhydrophobic coatings [51]. Copyright © 2021 Elsevier B.V. (**d**) Schematic diagram for the preparation of EHR-SNP, EHR-SNP-A, and nano-starch-based superhydrophobic coatings. Reprinted with permission from [52]. Copyright © 2021 American Chemical Society.

To exploit these features, several researchers have explored the fabrication of starch-based superhydrophobic materials. Ghasemlou et al. [49] fabricated nanohybrid superhydrophobic films by combining soft imprinting lithography and spin-coating techniques (Figure 5a). The composite matrix, comprising starch, polyhydroxyurethane, and cellulose nanocrystals (SPC), was engineered to replicate the hierarchical microstructure of lotus leaves. Starch functioned as a renewable substrate providing hydroxyl groups for covalent bonding with PDMS, while vinyl-modified silica nanoparticles enhanced nanoscale surface roughness. This hierarchical structure achieved a WCA of approximately 150° with an SA below 10°. Similarly, Ye et al. [50] applied ball-milled starch granules derived from rice, corn, and potato to form hierarchical roughness on hydroxypropyl methylcellulose (HPMC) films. Figure 5b shows the preparation process. The PDMS coating reduced surface energy, resulting in superhydrophobic surfaces with a WCA of 170.5° and an SA of 5.2°, alongside improved mechanical robustness due to the porous and stable nature of starch.

Tian’s team conducted a series of studies that further expanded the application range of starch-based superhydrophobic coatings. Tian et al. [51] prepared nanostarch-based coatings by enzymatically recrystallizing starch into nanoparticles, which were then combined with PDMS and applied via spray coating (Figure 5c). The resulting coral-like aggregates formed multiscale hierarchical structures, which, together with PDMS, imparted superhydrophobicity (WCA > 152°, SA < 9°). Building on this platform, Tian et al. [52] fabricated an eco-friendly, pH-responsive superhydrophobic coating by spray coating enzyme-hydrolyzed and recrystallized starch nanoparticles with PDMS (Figure 5d). The starch nanoparticles formed hierarchical micro/nanostructures, endowing the coating with a WCA exceeding 152.0° and an SA below 9.0°. Through the incorporation of anthocyanin-rich natural extracts, the material exhibited real-time colorimetric responsiveness for pH detection. Consequently, it maintained excellent water repellency and self-cleaning performance while demonstrating multifunctionality in smart packaging applications, particularly in monitoring food freshness.

Extending the functionality of starch-based materials, Tian et al. [53] prepared a superhydrophobic and superoleophilic adsorbent via the sol–gel impregnation of cetyltrimethoxysilane and ethyl orthosilicate in a starch gelatin matrix. The lyophilized starch provided structural support and porosity, while the generated hydrophobic groups and micro-nano roughness yielded a material with a high WCA (>153°), low SA (<8°), and excellent self-cleaning and antifouling properties. Subsequently, Tian et al. [54] developed a magnetically responsive superhydrophobic adsorbent by spray coating silylated porous starch/Fe_3_O_4_ hybrid micro-nanoparticles onto starch-based freeze-dried substrates. The porous starch matrix provided hierarchical surface roughness, yielding a WCA of >152.0° and an SA of <9.0°, while simultaneously functioning as an effective adsorbent. The incorporated Fe_3_O_4_ component imparted magnetic responsiveness, enabling remote actuation for the targeted removal of discrete oil spills from water surfaces.

Collectively, these investigations highlight the versatility and tunability of starch as a green substrate for the development of superhydrophobic materials. Through structural modification, nanostructuring, and functional integration with responsive or magnetic components, starch-based systems have demonstrated significant potential for applications in smart coatings, food packaging, environmental remediation, and oil–water separation technologies.

### 2.5. Silk Fibroin

Silk fibroin is derived from the cocoon of the Bombyx mori silkworm. It is a natural fibrous protein composed primarily of repeating glycine–alanine sequences, which confer high crystallinity and mechanical robustness. It is widely recognized in biomaterials research due to its excellent tensile strength, biocompatibility, biodegradability, and its ability to form stable nano- and microstructured assemblies. These characteristics, combined with its abundance of functional groups (particularly amine and hydroxyl groups), make silk fibroin a promising green substrate or structural matrix for the development of superhydrophobic surfaces. Depending on the fabrication process, silk fibroin can be processed into films, electrospun fiber mats, or porous sponges with intrinsic hierarchical morphology, which are amenable to further hydrophobic modification. Moreover, the compatibility of silk fibroin with both covalent and non-covalent surface functionalization strategies enables its integration with hydrophobic agents, such as silanes or nanoparticles, in order to construct durable superhydrophobic materials.

**Figure 6:**
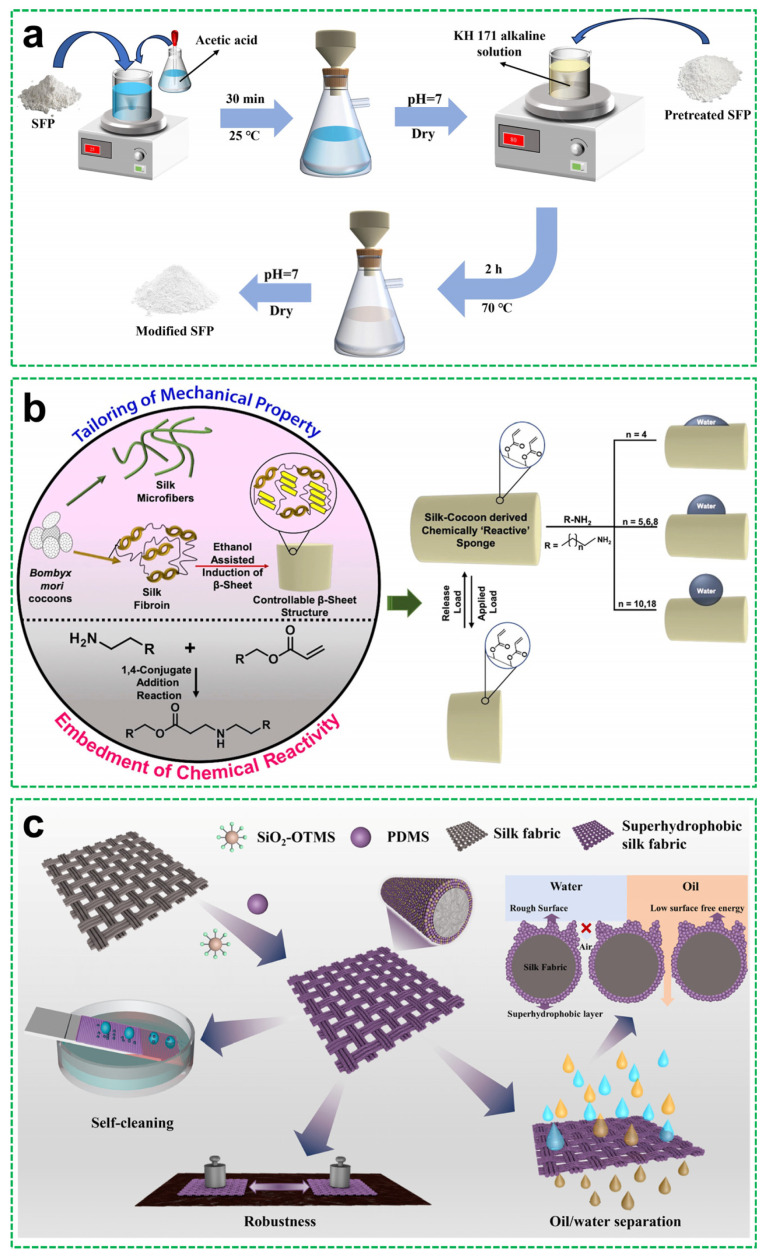
(**a**) Schematic of preparing KH-171-modified SFP [56]. Copyright © 2025 The Society of Powder Technology Japan. (**b**) Schematic Illustrating the Fabrication of Silk Cocoon-Derived Sponge with Controllable Mechanical Property and Chemical Reactivity. Reprinted with permission from [57]. Copyright © 2021 American Chemical Society. (**c**) Schematic illustration for the fabrication and functions of fluorine-free superhydrophobic silk fabric, surface superhydrophobicity mechanism, and oil–water separation mechanism [58]. Copyright © 2024 Elsevier B.V.

The superhydrophobic behavior of silk-fibroin-based materials is partly attributed to its intrinsic β-sheet crystalline domains, which are stabilized by intermolecular hydrogen bonds between peptide backbones. These hydrogen-bonded regions contribute to the stiffness and water resistance of the coating matrix. The interplay between ordered β-sheets and amorphous regions allows for tunable mechanical properties and compatibility with hydrophobic surface modifiers. Several studies have demonstrated the potential of silk fibroin in this context. Chen et al. [55] fabricated a superwetting stainless steel mesh by spray coating a composite of SiO_2_ and silk fibroin. Silk fibroin functioned as a hydrophilic scaffold, providing hydroxyl (–OH) and carboxyl (–COOH) groups that cross-linked with SiO_2_ via dehydration reactions. This covalent interaction constructed hierarchical micro/nanostructures, endowing the mesh with hydrophilic properties (WCA = 10°) and underwater superoleophobicity (oil contact angle = 150°). Consequently, the coated mesh achieved efficient oil–water separation. In another study, Huang et al. [56] synthesized superhydrophobic micro-nanostructured particles by modifying silk fibroin powder (SFP) with KH-171. Amine and hydroxyl groups on the silk fibroin backbone covalently bonded with silanol groups generated from KH-171 hydrolysis, forming polysiloxane oligomer nanoparticles (Figure 6a). The resultant hierarchical roughness endowed the material with a WCA of 161.8°, enabling oil–water separation at 99.1% efficiency.

Taking a more chemically tailored approach, Shome et al. [57] developed reactive silk fibroin sponges through a sequential process involving an ethanol-induced β-sheet crystallization and catalyst-free 1,4-conjugate addition between silk fibroin amine residues and acrylate-based cross-linkers (Figure 6b). The resulting polymeric network retained surface-reactive groups, enabling post-modification with octadecylamine to impart superhydrophobicity (WCA ≈ 161°). Here, silk microfiber reinforcement provided mechanical robustness, while the silk fibroin matrix served dually as a structural framework and reactive platform for functionalization, ultimately achieving efficient oil–water separation. Wei et al. [58] adopted a fluorine-free strategy to fabricate superhydrophobic silk fabrics by dip coating with octyltrimethoxysilane-modified SiO_2_ nanoparticles and PDMS. The silk fibroin substrate provided flexibility and conformal adhesion for the hydrophobic nanoparticles through physical interactions, forming robust micro/nanoporous hierarchical structures. This resulted in exceptional surface properties, including a WCA of 161.3 ± 1.5° and an SA of 5 ± 0.5°. The coated fabrics exhibited excellent durability, self-cleaning capability, and efficient oil–water separation performance across diverse environmental conditions; the underlying mechanisms are illustrated in Figure 6c.

These studies collectively highlight the versatility of silk fibroin as a green and functional matrix for constructing superhydrophobic surfaces. Its intrinsic mechanical strength, rich surface chemistry, and compatibility with various fabrication strategies, including silanization, nanoparticle incorporation, and cross-linking, enable the creation of durable, fluorine-free coatings with multifunctional properties. As a sustainable biopolymer, silk fibroin not only supports the development of environmentally benign superhydrophobic materials but also holds promise for applications requiring mechanical resilience, chemical tunability, and biocompatibility.

### 2.6. Other Green Materials

In addition to polysaccharides and proteins such as chitosan or silk fibroin, a broad spectrum of naturally derived lipids and biopolymers has been employed as substrates or functional components in superhydrophobic coatings. These include waxes, proteins, plant-derived polymers, and bio-resin materials that are not only renewable and biodegradable but also capable of contributing hydrophobicity or structural integrity to functional coatings.

**Figure 7:**
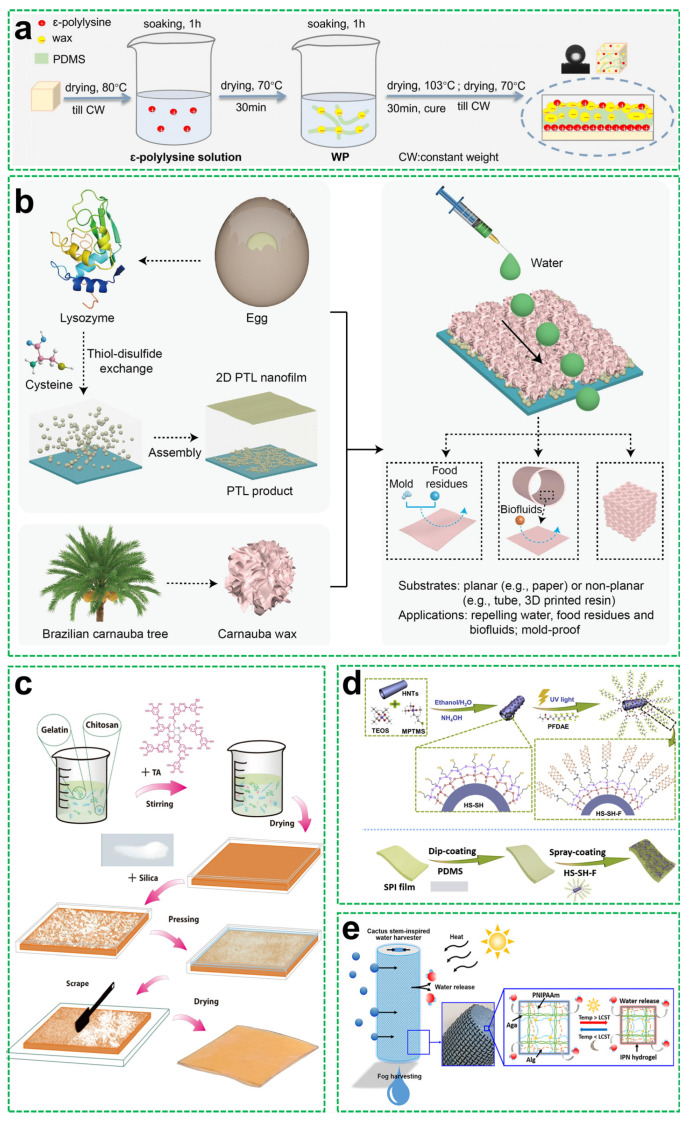
(**a**) Preparation process of superhydrophobic wood by the ε-polylysine/PDMS/wax composite system [59]. Copyright © 2024 Elsevier B.V. (**b**) Schematic illustration of the formation and applications of the all-natural superhydrophobic surface [60]. Copyright © 2020 Elsevier B.V. (**c**) Preparation flowchart of CGTS films [62]. Copyright © 2024 Elsevier B.V. (**d**) Schematic illustration of the synthesis route of HS-SH-F and HS-SH-F@SPI film [63]. Copyright © 2019 Elsevier B.V. (**e**) Schematic illustrations and photographs of the cactus-stem-inspired water harvesting system. IPN hydrogel consists of three networks to mimic the effective water absorption and long-term storage function of cactus stems with mucilage. Reprinted with permission from [64]. Copyright © 2019 American Chemical Society.

Natural waxes, such as beeswax and carnauba wax, are lipid-based substances secreted by plants and insects to create water-repellent surfaces in nature. Their long-chain esters inherently possess low surface energy, making them ideal hydrophobic agents. When used in coatings, these waxes—often in combination with plant-derived silica or polymer matrices—can achieve a WCA exceeding 150°. For instance, Cai et al. [59] fabricated durable superhydrophobic wood surfaces via layer-by-layer assembly of ε-polylysine (a cationic antimicrobial peptide), PDMS, and natural waxes (beeswax or carnauba) (Figure 7a). The negatively charged wood substrate enabled electrostatic adhesion enhancement through interactions with cationic ε-polylysine, while the wax components generated hierarchical micro-roughness on the wood’s inherent macrostructure. At an optimal ε-polylysine concentration of 2 g/L, this synergistic design achieved robust hydrophobicity (WCA > 150°, SA < 10°). Similarly, Li et al. [60] developed all-natural superhydrophobic coatings through cysteine-induced thiol–disulfide exchange, triggering phase transition in lysozyme to form microparticles. The carnauba wax coating generated hierarchical micro/nano-roughness while reducing surface energy, achieving a WCA >150° on diverse substrates, including paper and plastics. The coating exhibited exceptional resistance against food residues and blood contamination; the fabrication process and applications are illustrated in Figure 7b. Zhu et al. [61] developed self-healing hydrophobic cellulose films by infusing cellulose matrices with natural waxes (beeswax, candelilla wax, or carnauba wax) through alkali/urea dissolution followed by thermal annealing. During annealing, the wax components migrated to the surface, forming a continuous hydrophobic barrier layer. This yielded a WCA of ≈ 120° and tensile strength exceeding 120 MPa. The films demonstrated self-repair capability through repeated thermal annealing cycles to restore hydrophobicity after damage.

Protein-based systems also show significant promise. Gelatin, soybean protein isolate (SPI), and lysozyme, among others, can be cross-linked or hybridized with inorganic particles to fabricate robust, functional coatings. Yu et al. [62] reported superhydrophobic chitosan/gelatin/SiO_2_ (CGTS) films enhanced by tannic acid cross-linking; the preparation process is shown in Figure 7c. The incorporation of hydrophobic fumed silica particles and the hydrogen bonding network provided by tannic acid enabled strong mechanical integrity, and hydrophobic modification yielded a WCA of >150°, with antioxidant and antimicrobial properties. Liu et al. [63] prepared superamphiphobic coatings by depositing fluorinated halloysite nanotube–silica hybrid particles (HS-SH-F) onto PDMS-modified SPI films (Figure 7d). Tannic acid served both as a natural cross-linker and a reinforcing agent to improve coating stability and strength, resulting in an n-heptane WCA of 154.2° and an SA of 9.5°.

Beyond conventional biopolymers, natural resins and plant cuticle components are also gaining attention. For example, the cutin structural component of plant cuticles and shellac, a natural bio-resin secreted by insects, offer low surface energy and excellent film-forming capabilities. Although not yet extensively explored for superhydrophobic applications, these materials exhibit potential in specialized contexts. Lee et al. [64] developed a cactus-inspired water harvesting system by integrating a superhydrophilic interpenetrating polymer network (IPN) hydrogel composed of agarose, PNIPAAm, and alginate with a superhydrophobic copper mesh (SHPM). The SHPM was fabricated via chemical etching followed by surface modification with stearic acid, achieving a WCA of 157.6° (Figure 7e). This hybrid structure effectively mimicked the dual functionality of cactus stems (the water absorbing mucilage and evaporation-resistant cuticle), enabling both rapid water collection and reduced water loss under arid conditions. The resulting device exhibited a high water harvesting rate of 209 mg·cm^−2^·h^−1^, demonstrating its potential for efficient atmospheric water collection in desert-like environments.

In all these cases, the intrinsic chemical structures, such as long alkyl chains in lipids, β-sheet domains in proteins, or aromatic rings in polyphenols, contribute to low surface energy, while the hierarchical roughness necessary for superhydrophobicity is achieved either through natural texture or the incorporation of inorganic nanostructures (e.g., silica, titania, or alumina). These renewable materials are typically integrated using eco-friendly processes, such as solvent-free coating and dip coating, or mild cross-linking chemistries, avoiding the use of toxic fluorinated surfactants.

In general, these alternative green materials offer unique advantages in terms of sustainability, biocompatibility, and versatility. However, several challenges, such as inherent hydrophilicity (e.g., in polysaccharides or proteins), variability in natural feedstocks, and mechanical durability, remain. Therefore, synergistic design strategies, such as combining bio-based substrates with inorganic nanoparticles or using multifunctional natural cross-linkers, are essential in optimizing performance and ensuring the long-term applicability of these superhydrophobic coatings in environmental, biomedical, or packaging applications. To better understand the suitability of these natural materials for green superhydrophobic systems, Table 1 presents a comparative summary of representative biopolymers such as cellulose, chitosan, lignin, starch, and silk fibroin. The comparison highlights differences in mechanical strength, biodegradability, cost, and modifiability, offering practical guidance for material selection in sustainable coating design.

While Table 1 summarizes the biodegradability level of the major biopolymers used in green coatings, it is necessary to further highlight their decomposition behavior and environmental relevance. Many of the natural materials employed in green superhydrophobic surfaces, such as cellulose, chitosan, starch, and silk fibroin, are known to be biodegradable in natural environments. Under suitable conditions (e.g., the presence of moisture, oxygen, and microbial activity), these biopolymers can be enzymatically or microbially degraded into small, non-toxic molecules such as glucose, amino acids, CO_2_, and water. However, the hydrophobic surface modifications required to achieve superhydrophobicity, such as alkyl silane treatments, PDMS coating, or nanoparticle embedding, may reduce biodegradation rates by forming physical barriers or introducing recalcitrant functional groups.

Recent studies have indicated that biodegradation is highly sensitive to factors such as ambient temperature, pH, exposure duration, and substrate morphology. Moreover, few studies have systematically characterized the decomposition by-products or long-term fate of modified superhydrophobic biopolymers. This knowledge gap suggests a need for future research combining surface science, environmental toxicology, and biodegradation kinetics to ensure that green superhydrophobic coatings are not only bio-based but also environmentally benign throughout their life cycle.

## 3. Sustainable Fabrication Techniques of Superhydrophobic Surface

Achieving superhydrophobicity requires surfaces with micro-/nanoscale roughness and low surface energy. A critical research focus involves developing energy-efficient manufacturing methods utilizing benign solvents and ensuring scalability.

Compared to conventional techniques involving fluorinated compounds and harsh processing conditions, the manufacturing methods discussed in this section, including spray coating, dip coating, sol–gel processing, electrospinning, and laser ablation, offer significant environmental advantages. The selection of these methods was guided by their conformity to three core principles of green chemistry: (1) the utilization of environmentally friendly or renewable raw materials, (2) the minimization of toxic reagents or solvents, and (3) the enhancement of energy efficiency throughout the processing stages. Each technique was chosen not only for its ability to confer superhydrophobic properties but also for its alignment with the principles of green chemistry and sustainable engineering.

Consequently, this section categorizes and evaluates six principal manufacturing technologies, highlighting their applicability, environmental impact, and recent advancements in the development of green superhydrophobic surfaces. By fulfilling these criteria, these manufacturing technologies facilitate the sustainable and scalable development of superhydrophobic surfaces without compromising performance.

### 3.1. Spray Coating

Spray coating is a simple, energy-efficient, and scalable technique for fabricating superhydrophobic surfaces using green or bio-based formulations. By atomizing suspensions—typically water- or ethanol-containing functional materials—this method enables the rapid formation of micro/nanostructured textures on various substrates. The minimal equipment requirement, ranging from handheld airbrushes to automated systems, combined with mild drying conditions and low-toxicity solvents, makes spray coating particularly appealing for sustainable and large-area applications.

Depending on the functional requirements and composition of the spray formulation, spray-coated superhydrophobic surfaces have been successfully applied across multiple domains. In the field of oil–water separation, Li et al. [74] developed magnetic superhydrophobic filter paper via one-step spray coating of a dispersion containing epoxy resin, PDMS, Fe_3_O_4_, and SiO_2_ nanoparticles. The uniform deposition of hierarchical structures endowed the material with efficient oil–water separation capacity and self-cleaning performance while utilizing a low-temperature curing and ethanol-based process. Similarly, Zhang et al. [75] prepared a fluorine-free strategy by spraying candle soot, hexadecyltrimethoxysilane-modified attapulgite, and PDMS onto aluminum substrates. The resulting coatings exhibited outstanding water repellency and ice/corrosion resistance, demonstrating the viability of bioinspired, eco-friendly materials for functional interfaces.

**Figure 8:**
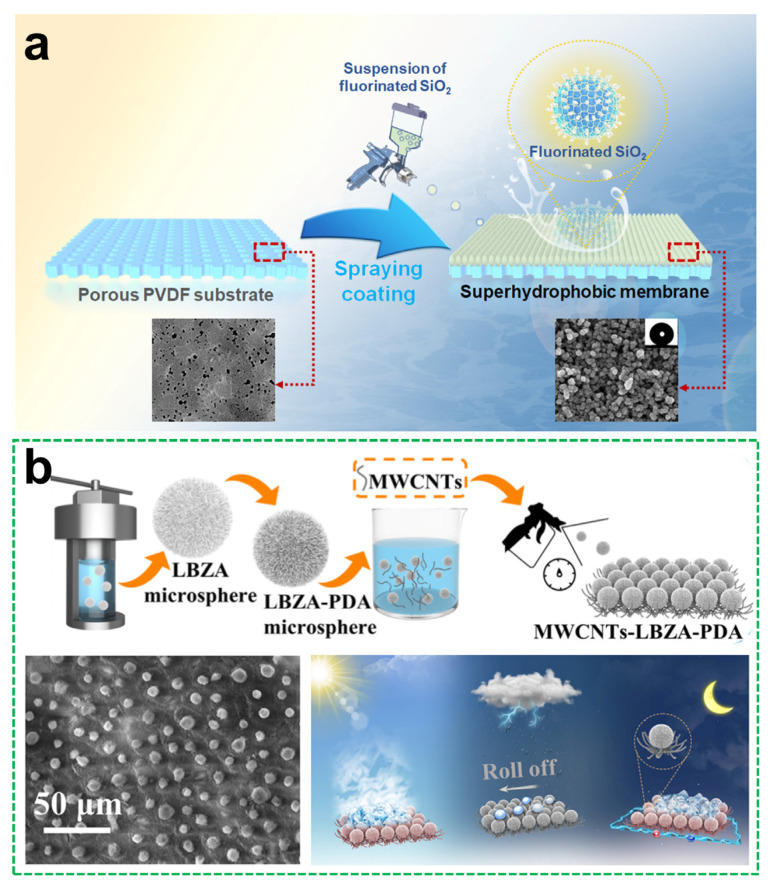
(**a**) Schematic diagram for the construction of superhydrophobic membranes by the spray coating of highly hydrophobic SiO_2_ nanoparticles [76]. Copyright © 2022 Elsevier B.V. (**b**) Experimental design. Schematic of the fabrication process for the coating overlayers, the ESEM image of the surface morphology of a lotus leaf, and a schematic for the anti-icing and deicing functions of the superhydrophobic overlayer in multiple scenarios. Reprinted with permission from [77]. Copyright © 2022 American Chemical Society.

Spray coating has also been applied to enhance membrane performance. Lin et al. [76] used this method to deposit fluorinated SiO_2_ nanoparticles onto polyvinylidene fluoride (PVDF) membranes, forming a durable rough surface that improved physicochemical stability and anti-wetting behavior in membrane distillation systems (Figure 8a). The incorporation of hierarchical structures significantly extended membrane lifespan and operational efficiency under harsh aqueous conditions.

Moving in another direction, corrosion protection and durability have driven the design of mechanically robust coatings. Zan et al. [77] combined layered zinc acetate microparticles and multi-walled carbon nanotubes, modified with polydopamine (PDA), into a photothermal- and electrothermal-responsive superhydrophobic surface (Figure 8b). The bioinspired hierarchical structure enabled efficient de-icing and anti-icing performance under various climatic stimuli, making it suitable for outdoor or aerospace applications. Meanwhile, Yang et al. [78] proposed a spray-deposited composite of micro-TiB_2_ and nano-SiO_2_, embedded in a PDMS@PU matrix, to form a multi-scale “hard particle–soft matrix” architecture. This design enhanced mechanical resilience, abrasion resistance, and long-term corrosion inhibition.

Across these diverse formulations, spray-coated surfaces consistently achieve high WCAs (often exceeding 160°), low SAs, and long-term environmental stability. The method’s compatibility with renewable materials, such as silica derived from rice husk ash, natural waxes, and PDA, further underscores its environmental benefits. Moreover, spray coating readily conforms to complex geometries and flexible substrates, enabling its integration into textiles, construction surfaces, packaging films, and wearable electronics. In summary, spray coating offers a versatile, low-impact approach for creating superhydrophobic surfaces aligned with green chemistry principles. Its operational simplicity, adaptability to scalable industrial processes, and ability to incorporate eco-friendly fillers make it a promising platform for future sustainable surface engineering. Continued research into binder selection, viscosity control, and post-deposition curing will further improve the uniformity, mechanical durability, and substrate adhesion of green superhydrophobic coatings.

### 3.2. Dip Coating

Dip coating is a simple, low-energy, and environmentally benign technique for fabricating superhydrophobic surfaces. Unlike spray coating, which relies on aerosolized deposition, dip coating immerses substrates into liquid formulations—typically aqueous or bioethanol-based dispersions—followed by controlled withdrawal in order to achieve uniform film formation. This method offers excellent control over film thickness and conformal coverage, making it particularly advantageous for coating fibrous or porous substrates such as textiles, sponges, and natural fabrics. Moreover, it minimizes material waste and can be performed under ambient conditions, making it highly compatible with green manufacturing practices. Dip-coated superhydrophobic materials are often constructed by depositing bio-based polymers, waxes, or nanoparticles onto substrates to create hierarchical surface roughness and to reduce surface energy. This method supports both single- and multi-step fabrication routes, enabling the integration of multifunctional properties beyond water repellency.

**Figure 9:**
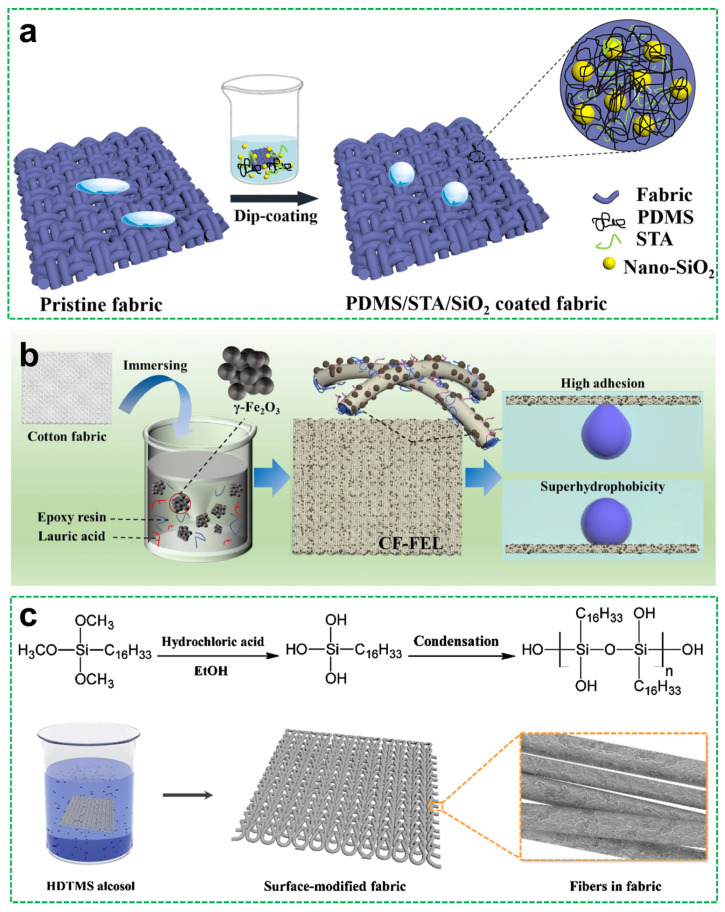
(**a**) Fabrication scheme of the superhydrophobic coating on the fabric [80]. (**b**) Schematic illustration for the preparation process of CF-FEL [81]. Copyright © 2024 Elsevier B.V. (**c**) Chemical structure and hydrolysis-condensation procedure of HDTMS; the schematic diagram of the calcium alginate fabric treated by HDTMS alcosol [82]. Reproduced with permission from Springer Nature.

Dip coating is especially effective in modifying textile-based and sponge-like materials. Chen et al. [79] fabricated flame-retardant and electrically conductive PU sponges via dip coating in a PDA/reduced graphene oxide (RGO) suspension, followed by grafting with perfluorooctyltriethoxysilane (PFOTES). The resulting sponge exhibited superhydrophobicity, self-cleaning behavior, and enhanced electrical properties due to the uniformly distributed nano-roughness and low-surface-energy components. Liu et al. [80] employed a one-step dip-coating method to modify polyester fabrics using a PDMS–stearic acid–nano–SiO_2_ dispersion in ethyl acetate (Figure 9a). The dip-coated composite provided the fabrics with durable superhydrophobicity and excellent self-cleaning capability, benefiting from both chemical hydrophobization and hierarchical micro/nano surface features.

Dip coating also enables the fabrication of multifunctional coatings through the incorporation of active fillers. For instance, Wang et al. [81] developed magnetic and thermally stable cotton fabrics by dip coating in a suspension of *γ*-Fe_2_O_3_ nanoparticles, epoxy resin, and lauric acid; the preparation process is shown in Figure 9b. The *γ*-Fe_2_O_3_ imparted flame retardancy and magnetic responsiveness, while the epoxy matrix ensured good adhesion and structural integrity. In another example, Zheng et al. [82] created flame-retardant alginate fabrics by dip coating in hexadecyltrimethoxysilane (HDTMS) alcohol; Figure 9c shows the preparation process and application of superhydrophobic fabrics. The in situ hydrolysis and condensation of HDTMS formed micro/nanostructured hydrophobic layers, effectively maintaining the native flame-retardant properties of calcium alginate while adding self-cleaning capability.

Overall, dip coating stands out as a scalable and eco-friendly method for engineering superhydrophobic surfaces with diverse functionalities. Its compatibility with natural or biodegradable components makes it an attractive candidate for sustainable material development. Future research may focus on multilayer or gradient dip-coating strategies, solvent reuse and recycling, and the integration of smart functionalities such as stimuli-responsive or self-healing behavior. Additionally, coupling dip coating with other green processing methods may further enhance adhesion and durability on complex substrates.

### 3.3. Sol–Gel Methods

The sol–gel method, based on the hydrolysis and condensation of metal alkoxides or silica precursors, is a versatile wet chemical route for fabricating superhydrophobic surfaces. This technique enables the formation of nanoporous or nanostructured silica networks at relatively low temperatures, typically under mild conditions, using green solvents such as water or ethanol. From a sustainability perspective, sol–gel processes are inherently advantageous: they require minimal energy input, use low-toxicity reagents, and are compatible with scalable deposition techniques including dip coating, spray coating, and spin coating. The resulting coatings often exhibit the tunable surface topographies that are essential for achieving hierarchical roughness and low surface energy.

**Figure 10:**
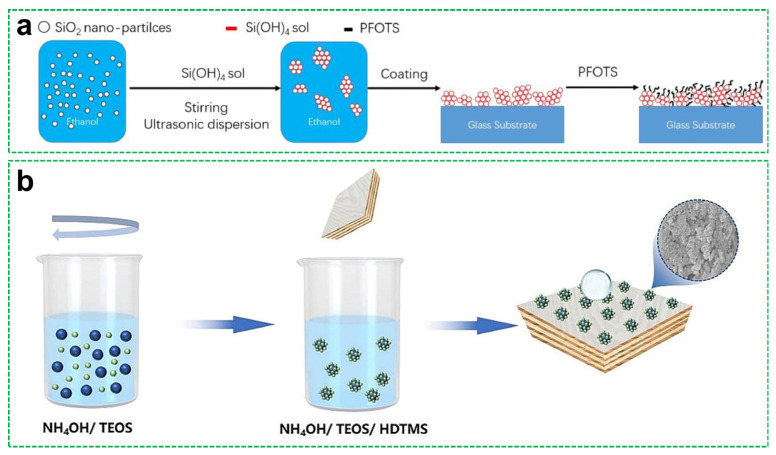
(**a**) Schematic illustration of the preparation process [84]. Copyright © 2021 Elsevier B.V. (**b**) Flow chart of the preparation of nanocellulose-based superhydrophobic wood-based panels by the sol–gel method [86].

Several studies have demonstrated the effectiveness of sol–gel methods in fabricating durable and functional superhydrophobic coatings. For instance, Hashjin et al. [83] designed highly robust coatings by alternating acidic (pH 4) and basic (pH 8) sol layers composed of tetraethoxysilane (TEOS), methyltriethoxysilane (MTES), and triethoxyoctylsilane, along with fumed silica nanoparticles. This alternating pH strategy facilitated the formation of hierarchically structured silica networks with enhanced cross-linking and improved substrate adhesion. The resulting coatings exhibited superior mechanical strength and weather resistance. In another approach, Ke et al. [84] focused on achieving transparency alongside superhydrophobicity; Figure 10a shows the preparation process and the application of superhydrophobic coatings. SiO_2_ nanoparticles were dispersed into a TEOS-based sol and the surface was functionalized with PFOTES. By optimizing the ratio between TEOS and nanoparticles, Ke et al. balanced the competing requirements of surface roughness (to support a WCA of 154°) and high optical transmittance (with a less--than-1% reduction compared to bare glass), demonstrating the adaptability of sol–gel chemistry for optical applications. Mahadik et al. [85] employed a dip-coating strategy to prepare multifunctional coatings on glass substrates using MTES-derived sols. Surface modification with trimethylchlorosilane (TMCS) yielded a micro/nanostructured surface exhibiting a WCA of 153 ± 3°. The coatings also demonstrated notable thermal stability, with only 0.429 wt% weight loss, attributed to the well-developed organosilane network that formed during controlled sol–gel deposition.

Beyond mineral substrates, the sol–gel approach has been extended to biomass-based materials. Yao et al. [86] integrated sol–gel chemistry with nanocellulose-based films by immersing CNF/PVA-coated wood panels in a TEOS/HDTMS solution (Figure 10a). This treatment generated a rough, silica-based surface with low surface energy, achieving a high WCA (166.8°) and self-cleaning performance. The success of this hybrid strategy highlights the compatibility of sol–gel techniques with bio-derived substrates in developing fully green functional coatings.

Despite its promise, the sol–gel method still faces several practical challenges. Achieving uniform coatings with consistent micro/nano-scale features across diverse substrates requires precise control over process parameters, such as sol composition, pH, aging time, and drying conditions. Furthermore, improving the mechanical robustness and long-term durability of sol–gel-based coatings—particularly under outdoor or abrasive conditions—remains a critical research direction. These challenges are especially relevant when striving to maintain environmental compatibility by avoiding fluorinated agents or toxic cross-linkers.

### 3.4. Layer-by-Layer (LbL) and Self-Assembly

In contrast to sol–gel approaches, which construct nanostructured coatings through chemical condensation, LbL assembly and self-assembly techniques achieve ordered nanostructures via non-covalent interactions such as electrostatics, hydrogen bonding, and hydrophobic forces. These strategies are typically conducted under mild conditions (room temperature, aqueous solvents), aligning well with green chemistry principles. Moreover, they offer molecular-level control over surface architecture, enabling the rational design of hierarchical structures without the need for toxic reagents or complex equipment.

LbL assembly builds up thin films by alternating the deposition of oppositely charged materials (e.g., polyelectrolytes or nanoparticles), while self-assembly often relies on the spontaneous organization of amphiphilic or reactive species into functional architectures. Though both methods may be limited by relatively slow deposition rates or small-scale throughput, they offer exceptional flexibility in tuning surface morphology, wettability, and functionality.

**Figure 11:**
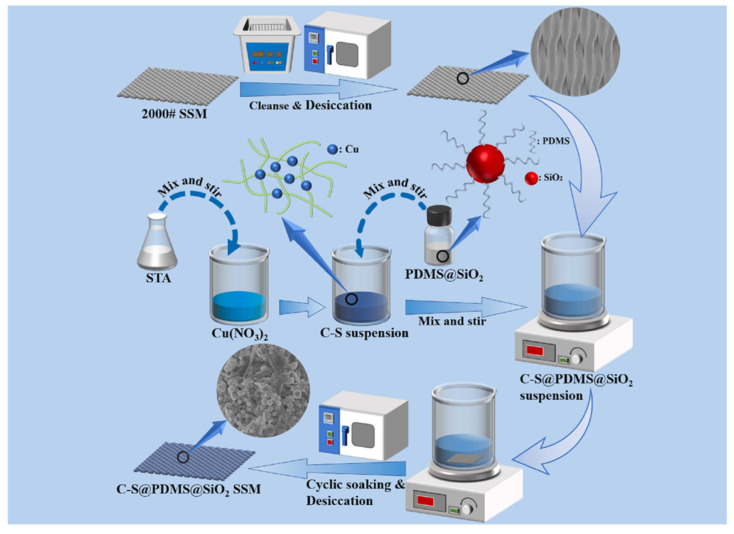
Schematic diagram of the preparation of C-S@PDMS@SiO_2_ SSM [87]. Copyright © 2024 Elsevier Ltd.

Self-assembly techniques have shown great promise in designing superhydrophobic surfaces for efficient oil–water separation. Li et al. [87] reported the preparation of fluorine-free superhydrophobic coatings on stainless steel mesh using a combination of self-assembly and spray coating, as shown in Figure 11. The spontaneous organization of copper stearate and hydrophobic SiO_2_ nanoparticles formed micro/nanostructures, while the spray technique ensured uniform film deposition. The resulting coating exhibited excellent oil–water separation efficiency (>99.78%). Similarly, Yang et al. [88] employed the self-assembly of iron ions with 1,3,5-benzenetricarboxylic acid (H_3_BTC) to form a metal–organic framework (MOF) network on cotton fabric. Subsequent PDMS modification rendered the material superhydrophobic, achieving ultra-high flux (32,527 L·m^−2^·h^−1^) in oil–water separation applications. These examples highlight the compatibility of self-assembly techniques with bio-based substrates and scalable processing.

**Figure 12:**
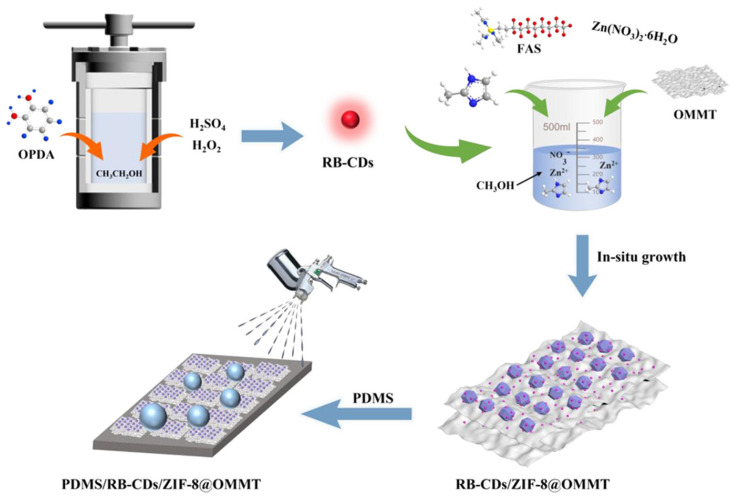
Schematic Representation of the Fabrication of the Superhydrophobic Coating. Reprinted with permission from [89]. Copyright © 2023 American Chemical Society.

Beyond separation, self-assembled coatings have been engineered for multifunctional and photothermal applications. Shi et al. [89] developed fluorescent superhydrophobic coatings through in situ self-assembly of ZIF-8 nanoparticles and carbon dots on montmorillonite clay, followed by PDMS overcoating (Figure 12). The resulting hierarchical structures exhibited strong water repellency, self-cleaning capability, and photothermal conversion under light irradiation. In a related approach, Zhu et al. [90] utilized the evaporation-induced self-assembly of PVDF clusters to fabricate superhydrophobic coatings with anti-icing functionality. The solvent-driven aggregation process produced microstructured surfaces that reduced ice adhesion and delayed freezing, making them suitable for cold-weather or aerospace applications.

**Figure 13:**
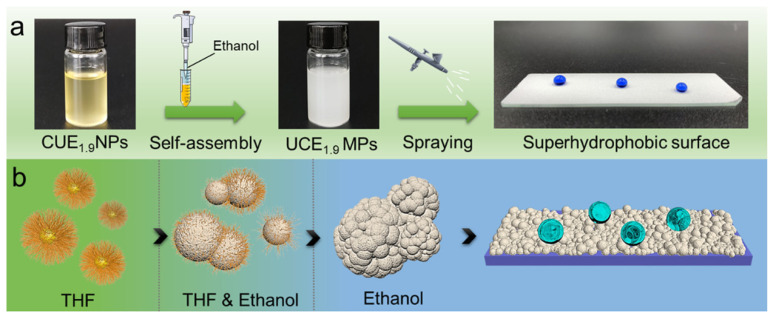
(**a**) Photograph of the preparation process of the superhydrophobic surface. (**b**) Schematic of the self-assembled MP formation via the addition of ethanol in the THF suspension of UCE_1.9_. Reprinted with permission from [91]. Copyright © 2021 American Chemical Society.

Self-assembly has also been employed in developing structurally stable coatings for harsh environments. Tang et al. [91] reported the solvent-exchange-induced self-assembly of undecenoate cellulose ester (UCE_1.9_) into micro/nanoparticles, forming cellulose-based coatings with high acid/alkali resistance and thermal stability. Figure 13 shows the preparation of the UCE_1.9_-based superhydrophobic surface. Meanwhile, Lee et al. [92] synthesized marine antifouling superhydrophobic materials by CO_2_ mineralization and self-assembly of calcium carbonate nanoparticles derived from seawater, which formed flower-like hierarchical structures with low surface energy. Ning et al. [93] conducted a comprehensive review of self-assembly methods—including self-assembled monolayers, Langmuir–Blodgett deposition, and LbL assembly—highlighting how weak intermolecular forces enable the formation of ordered micro/nanostructures. These structures, in turn, facilitate the attainment of Cassie–Baxter wetting states through hierarchical roughness and low surface energy.

Although LbL assembly can be time-consuming and is typically limited to smaller substrates, it remains a valuable technique for constructing multi-layered architectures without the use of organic solvents. Similarly, aqueous self-assembly methods, involving amphiphilic biomolecules or block copolymers, offer green and scalable routes to superhydrophobic surface engineering. In practice, these methods are often combined with other eco-friendly techniques, e.g., LbL deposition followed by wax impregnation or spray coating to enhance performance while maintaining environmental compatibility [59].

### 3.5. Electrospinning

In contrast to solution-based techniques such as dip coating or sol–gel processing, which typically yield uniform, thin films, electrospinning offers a unique strategy for fabricating superhydrophobic surfaces by generating three-dimensional fibrous architectures. This method allows precise control over surface morphology, enabling the construction of the multiscale roughness essential for superhydrophobicity. More importantly, electrospinning is compatible with a wide range of biodegradable polymers and green solvents, making it an environmentally friendly approach for developing functional coatings.

Electrospun materials have shown particular promise in oil–water separation, where the combination of high porosity, tunable surface chemistry, and capillary effects provides efficient separation performance. For example, Liu et al. [94] fabricated flexible polyimide nanofibrous membranes through electrospinning, followed by surface modification using PDA and polytetrafluoroethylene (PTFE) nanoparticles. The dual-scale roughness and low surface energy imparted by the treatment enabled effective oil–water separation with superoleophilic and superhydrophobic characteristics. Similarly, Zhang et al. [95] reviewed advances in nanofibrous membranes for emulsion separation, highlighting how tailored electrospun structures—achieved via multi-spinneret systems or template-assisted patterning—enhance selectivity and flux by adjusting fiber diameter, surface energy, and porosity.

Beyond separation, electrospinning also facilitates the fabrication of multifunctional superhydrophobic composites by incorporating functional nanoparticles during fiber formation. Wang and Li [96] demonstrated this approach by electrospinning a blend of polystyrene, poly(acrylonitrile)-poly(vinylidene fluoride), and PDMS containing Fe_3_O_4_ and TiO_2_ nanoparticles. The resulting membrane integrated magnetic and UV-resistant properties while maintaining high WCAs and excellent reusability, demonstrating the potential of electrospinning for producing stimuli-responsive, reusable materials in environmental or sensing applications.

**Figure 14:**
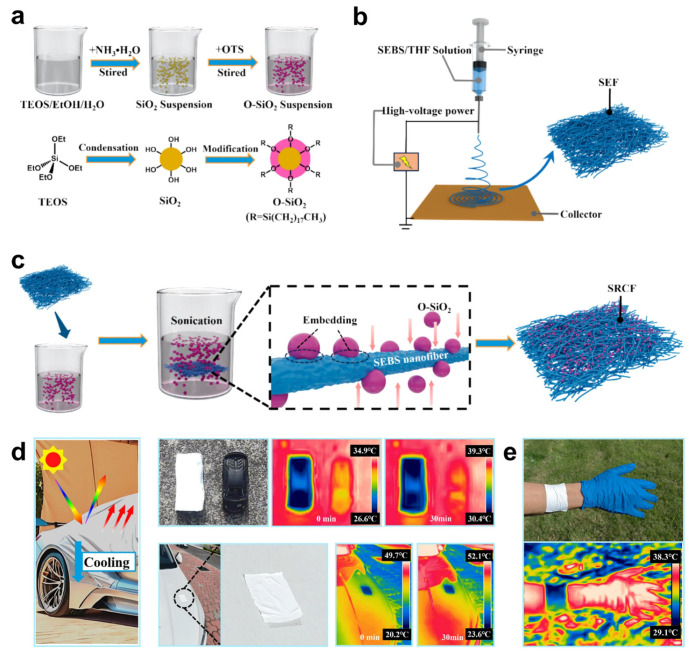
The fabrication of SRCF. (**a**) Schematic diagram of O-SiO_2_ preparation. (**b**) Illustration of the SEF fabrication process. (**c**) Diagram of the fabrication process of SRCF. (**d**) Application of the SRCF as a car clothing. (**e**) Photograph of bare skin covered with SRCF and its thermal IR image under sunlight exposure [97]. Copyright © 2025 Elsevier B.V.

Electrospun superhydrophobic materials have also been leveraged for thermal management and environmental regulation. Xu et al. [97] developed radiative cooling fabrics (SRCF) by electrospinning styrene–ethylene/butylene–styrene (SEBS) and subsequently depositing OTS-modified silica nanoparticles. Figure 14 shows the fabrication process and application of SRCF. The coating exhibited high solar reflectance, strong thermal emissivity, and durable water repellency, offering a passive, eco-friendly strategy for thermal control in textiles or architectural surfaces.

In summary, electrospinning represents a flexible and scalable method for engineering green superhydrophobic surfaces, particularly when fibrous morphology, multifunctionality, or surface tunability is required. Current research trends emphasize the integration of biodegradable polymer matrices, solvent recovery systems, and the incorporation of smart functionalities (e.g., self-healing, stimuli responsiveness) to expand the scope of applications in sustainable surface engineering.

### 3.6. Laser Techniques

As a dry, solvent-free alternative to solution-based approaches, laser processing represents a top-down strategy for fabricating superhydrophobic surfaces through direct surface texturing. Unlike bottom-up methods such as electrospinning or dip coating, laser techniques modify substrate morphology with high spatial precision, enabling the creation of hierarchical roughness without chemical waste or post-processing. This inherently “green” process eliminates the use of hazardous solvents and is compatible with a wide range of materials, including metals, polymers, and glass.

Direct laser texturing forms the foundation of many superhydrophobic coatings, where ultrashort pulse lasers, such as femtosecond or picosecond lasers, are used to sculpt microgrooves, nanoridges, or multiscale topographies. These features promote the Cassie–Baxter wetting state by minimizing the solid–liquid contact area. For example, Chakraborty et al. [98] employed pulsed laser ablation of PDMS-coated substrates (Polymethyl Methacrylate, glass, and aluminum), generating in situ micro/nanoparticles that redeposited onto the surface to create hierarchical roughness. This one-step, additive-free process achieved WCAs above 150° and roll-off angles below 3°, all without chemical modification. Similarly, Radhakrishnan et al. [99] used picosecond lasers to create dense micro/nano-bump structures on TiN surfaces. Subsequent exposure to ambient air enabled the passive adsorption of airborne hydrocarbons, inducing low-adhesion superhydrophobicity.

Another widely explored strategy is utilizing natural adsorption following laser structuring to eliminate the need for fluorinated chemicals. Tong and Xiong [100] concluded that the direct laser texturing of metallic substrates, such as aluminum and stainless steel, can induce wetting transitions via roughness tuning, while ambient organic species passively lower the surface energy. This passive, environmentally benign route avoids synthetic coatings altogether, making it especially attractive for large-scale or field applications.

**Figure 15:**
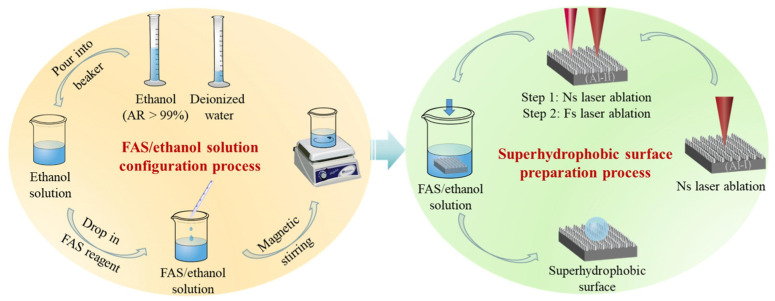
Schematic diagram of the superhydrophobic surface fabrication process on Al substrates [102]. Copyright © 2023 Elsevier Ltd.

In addition, hybrid-laser-assisted techniques have been developed to combine structural precision with chemical versatility. Xu et al. [101] introduced a laser-enhanced electrodeposition method in which localized laser heating accelerated the reduction of copper ions, forming sponge-like micro/nanostructures later functionalized with PFDTES. The result was a highly water-repellent surface (contact angle~155°) with anti-icing capabilities. In another example, Yang et al. [102] applied sequential nanosecond–femtosecond (ns–fs) laser processing to generate complex multiscale patterns on aluminum, followed by silanization; the preparation process is shown in Figure 15. This approach significantly improved both corrosion resistance and electrochemical durability.

While laser techniques offer unmatched precision, they also face challenges in scalability, energy consumption, and equipment cost. Processing large-area or flexible substrates remains a limitation due to the point-by-point nature of laser ablation and the need for high-end optical systems. Nevertheless, advances in low-power, portable laser systems and hybrid methods, such as coupling laser texturing with plasma or waterjet processing, are rapidly improving throughput and broadening applicability.

In summary, laser-based methods provide a precise, waste-free, and chemically benign route to engineer robust superhydrophobic surfaces. Their ability to pattern micro/nanostructures directly onto diverse substrates, without the use of solvents or fluorinated additives, positions them as a compelling strategy in the sustainable manufacturing of high-performance water-repellent materials. Ongoing innovations in system integration and energy efficiency will further enhance their viability for industrial-scale, eco-conscious applications.

**Figure 16:**
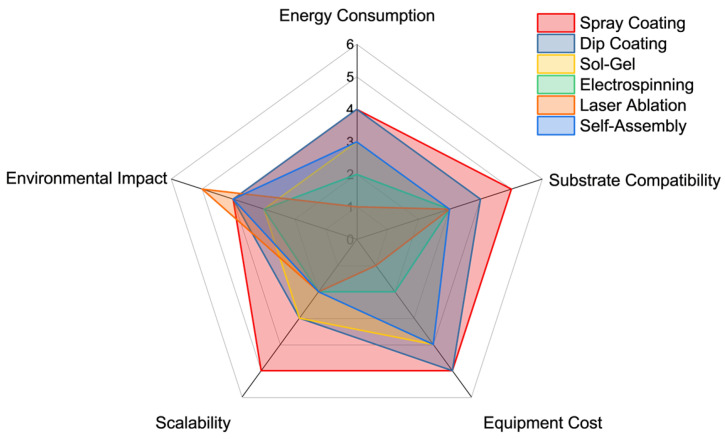
Comparative radar chart of sustainable fabrication techniques for superhydrophobic surfaces.

The sustainable fabrication of superhydrophobic materials has rapidly evolved beyond traditional fluorinated coatings and energy-intensive processes. Across a diverse range of scalable techniques—including sol–gel synthesis, spray coating, dip coating, electrospinning, laser texturing, and self-assembly—researchers have demonstrated that environmental compatibility need not come at the expense of functionality. Each method offers distinct advantages: sol–gel routes enable chemically bonded coatings with tunable surface energy; spray- and dip coating provides simplicity, scalability, and compatibility with natural dispersions; electrospinning affords large-area nanofiber networks with high porosity and functional versatility; laser techniques allow solvent-free microstructuring with precision; bottom-up strategies like LbL and self-assembly offer molecular-level control using water-based systems. To facilitate a clearer comparison between fabrication approaches, a summary diagram is presented in Figure 16. This comparative overview evaluates the six primary sustainable techniques based on typical criteria such as energy requirements, substrate compatibility, processing cost, environmental safety, and potential for scale-up.

It is worth noting that many of these techniques can be adapted to use biodegradable polymers, bio-derived particles, or aqueous systems, thereby reducing reliance on toxic solvents and non-renewable components. Furthermore, hybrid approaches that integrate multiple techniques (such as combining LbL assembly with spray deposition or embedding nanoparticles within electrospun mats) can enhance durability, reusability, and multifunctionality (e.g., anti-icing, photothermal conversion, oil–water separation).

Another noteworthy point is that, during the fabrication of superhydrophobic coatings, coating thickness and porosity play a critical role in defining the functionality and reliability of superhydrophobic surfaces. A sufficient coating thickness is required to maintain hierarchical roughness and air retention capacity, both of which are essential for achieving high water contact and low sliding angles [103]. However, overly thick coatings may suffer from reduced transparency, increased brittleness, and detachment from the substrate under mechanical stress. On the other hand, porosity promotes air trapping and water repellency but can also weaken abrasion resistance, reduce barrier integrity, or create pathways for contaminant penetration [104].

Different fabrication methods offer varying degrees of control over these parameters. In dip coating, thickness is modulated by adjusting withdrawal speed, solution viscosity, and ambient drying conditions. Spray coating enables control through spray duration, nozzle-to-substrate distance, and solvent composition. Sol–gel techniques rely on precursor concentration and gelation time, with post-deposition drying or annealing playing a crucial role in adjusting both porosity and film density. Electrospinning and phase separation naturally produce porous fibrous mats, where thickness and morphology are influenced by polymer concentration, voltage, and environmental humidity. Self-assembled monolayers, though thin and uniform, may not provide the required roughness for durable superhydrophobicity without additional structuring.

Despite significant progress, challenges remain in terms of scaling up to industrial-level production, maintaining long-term environmental resistance, and achieving cost-effectiveness using only green feedstocks. Nonetheless, the toolbox of sustainable fabrication techniques continues to expand, paving the way for truly eco-friendly and high-performance superhydrophobic materials across sectors from textiles to energy systems.

## 4. Applications and Performance of Green Superhydrophobic Materials

Superhydrophobic coatings have demonstrated remarkable multifunctionality across a wide spectrum of applications, driven by their unique surface wettability, micro/nanostructures, and chemical compositions. In self-cleaning and antifouling contexts, these coatings effectively remove contaminants and resist microbial adhesion, thereby extending the service life of surfaces. Anti-corrosion and anti-icing applications benefit from the air barrier and water-repellent effects, which reduce ion penetration and ice nucleation, respectively, even under harsh environmental conditions. In the field of oil–water separation, superhydrophobic materials—particularly those derived from biomass—enable high-efficiency separation and environmental sustainability. In food packaging and biomedical domains, the integration of biocompatibility, antibacterial functions, and low toxicity broadens their utility in sensitive settings. Furthermore, advancements in drag reduction, optical clarity, and self-healing functionalities expand their prospects in the marine, textile, and optical industries.

### 4.1. Self-Cleaning Surfaces

Superhydrophobic surfaces—characterized by their ability to cause water droplets to bead up and roll off—exhibit an inherent self-cleaning mechanism that effectively removes adhered dust, particles, and contaminants. This phenomenon, widely known as the lotus leaf effect, mimics natural surfaces such as insect wings and plant leaves, where the combination of micro-/nano-roughness and low surface energy allows for water mobility and pathogen inhibition. Such passive cleaning not only reduces maintenance frequency but also aligns well with sustainable, low-detergent strategies.

**Figure 17:**
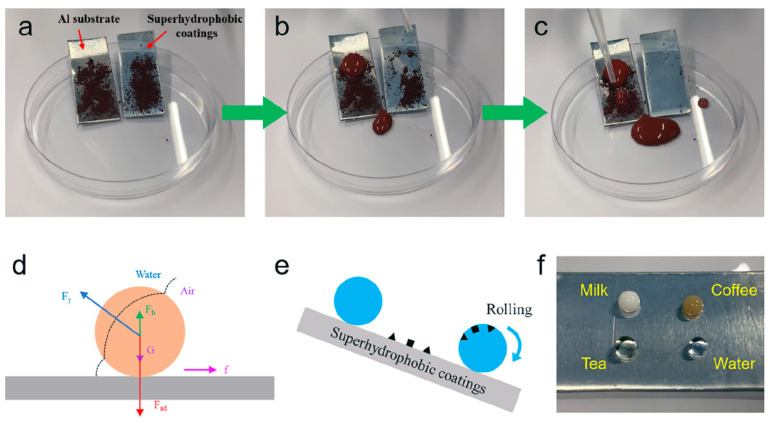
(**a**–**c**) Self-cleaning property of Al substrate and superhydrophobic ZIF-8@SiO_2_/PDMS coatings. (**d**) Schematic diagram illustrating the force of a dust particle exerted on the superhydrophobic ZIF-8@SiO_2_/PDMS coating. (**e**) Schematic representation of the self-cleaning process. (**f**) Image of different liquid droplets placed on the superhydrophobic ZIF-8@SiO_2_/PDMS coating. Reprinted with permission from [105]. Copyright © 2021 American Chemical Society.

Inspired by nature, researchers have engineered a wide range of artificial superhydrophobic coatings with enhanced self-cleaning capabilities. For instance, Jin et al. [105] developed a ZIF-8@SiO_2_/PDMS composite coating via spin coating, which featured hierarchical micro/nanostructures that enabled effective removal of dust through rolling water droplets; Figure 17 shows the self-cleaning properties and mechanism of the coating. This coating also demonstrated excellent corrosion resistance in a 3.5 wt% NaCl solution, suggesting multifunctionality. Similarly, Li et al. [106] prepared a fluorine-free ZnO@PDMS superhydrophobic surface using spray coating. The resulting micro/nano composite structure not only facilitated the removal of methylene blue particles but also exhibited strong antifouling performance against liquids such as milk and tea.

To further enhance durability under harsh environmental conditions, self-healing superhydrophobic surfaces have been developed. Li et al. [107] fabricated robust self-healable superhydrophobic fabrics via in situ diazonium radical polymerization, constructing hierarchical Cu_2_O/Cu_7_Cl_4_(OH)_10_H_2_O micro/nanostructures grafted with CF_3_Ph groups on cotton fabric. The fabric exhibits a WCA of 161.4° and an SA of 1.4°, enabling self-cleaning by rolling water droplets to remove dye powders (e.g., methylene blue, rhodamine B) through the Cassie–Baxter mechanism, and maintains this property after 20 laundering cycles and 1500 abrasion cycles. Likewise, Chen et al. [108] employed in situ diazonium radical polymerization to construct robust, self-healing superhydrophobic fabrics featuring hierarchical PDMS/ODA-PDA@PI microstructures. These fabrics effectively repelled water and oil contaminants and were suitable for applications such as oily wastewater purification. Ma et al. [109] also designed durable electrospun PI membranes with PDMS/ODA and PDA coatings. Their multi-scale roughness allowed for efficient repulsion of water droplets and the removal of particulate matter during oil–water separation, further highlighting the potential of such materials in environmental remediation.

Importantly, the incorporation of biodegradable or bio-derived components into self-cleaning coatings represents a promising approach towards greener material lifecycles. As these coatings eventually degrade or wear out, minimizing their environmental footprint through the use of eco-friendly components will be crucial for widespread adoption in sustainable technologies.

### 4.2. Oil–Water Separation

**Figure 18:**
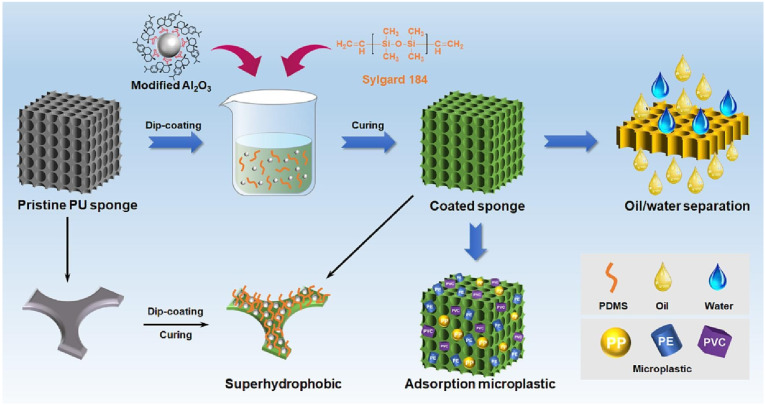
Schematic illustration of the preparation and application of the PDMS/DR-Al_2_O_3_ sponge for oil–water separation and microplastic removal [110]. Copyright © 2023 Elsevier B.V.

As a prominent application of superhydrophobic materials, oil–water separation leverages the contrasting wetting properties of engineered surfaces to achieve selective permeation. In particular, superhydrophobic–superoleophilic interfaces can allow oil to be absorbed or pass through, while simultaneously repelling water. This unique behavior, combined with advances in green fabrication, has enabled the development of environmentally friendly materials for the efficient separation of oil–water mixtures and emulsions. In particular, the incorporation of biomass-derived or biodegradable components aligns with the growing emphasis on sustainability in environmental remediation. Liu et al. [110] developed an eco-friendly superhydrophobic PU sponge via dip coating with PDMS and dehydroabietic-acid-grafted Al_2_O_3_ nanoparticles, as shown in Figure 18. The sponge exhibited high oil absorption capacity and efficiently separated both free-phase and emulsified oil–water systems. Moreover, it demonstrated the potential to remove microplastics through synergistic capillary forces and non-covalent interactions. Guo et al. [111] fabricated a high-performance superhydrophobic membrane by spraying a mixture of multi-walled carbon nanotubes (MWCNTs) and epoxy resin onto stainless steel mesh. The resultant surface exhibited ultra-high permeation flux for various oils (e.g., up to 77,023.6 L·m^−2^·h^−1^ for carbon tetrachloride), while maintaining separation efficiencies above 99.5% for water-in-oil emulsions. Yu et al. [112] prepared superhydrophobic fabrics through a one-step coating method using polyethyleneimine (PEI), trimethoxysilane propyl acrylate (TMSPA), SiO_2_ nanoparticles, and dodecyltrimethoxysilane (DTMS). These fabrics achieved a separation efficiency of 98.49% after 18 repeated cycles and were successfully applied in oil spill remediation through the use of a miniaturized, water-repellent “collection boat.”

Abu-Thabit et al. [113] reviewed the fabrication strategies for superhydrophobic/superoleophilic nanohybrid sponges composed of biodegradable polymers such as PU, melamine, or cellulose. These sponges, typically prepared via dip coating or sol–gel routes, and functionalized with PDMS, metal oxides, or carbon-based nanoparticles, have shown great promise in separating both immiscible and emulsified oil–water systems. Importantly, their biocompatibility supports the regeneration of sorbents and, in some cases, the reuse of recovered oil, such as in biofuel applications.

Overall, these advancements highlight the practicality of green superhydrophobic materials for oil–water separation, offering scalable, recyclable, and eco-friendly alternatives to conventional approaches. Future research may focus on enhancing long-term durability, fouling resistance, and integration into smart or responsive separation systems.

### 4.3. Anti-Corrosion

Metal corrosion, especially under humid, saline, or marine conditions, remains a pervasive challenge across industrial sectors. Superhydrophobic coatings, by virtue of their low surface energy and ability to entrap air pockets, act as effective barriers that impede the diffusion of corrosive species such as Cl^−^ and OH^−^ ions. These coatings not only suppress electrochemical reactions on the metal surface but also offer auxiliary functionalities, such as anti-scaling, deicing, and antifouling, all of which are increasingly critical in harsh service environments.

**Figure 19:**
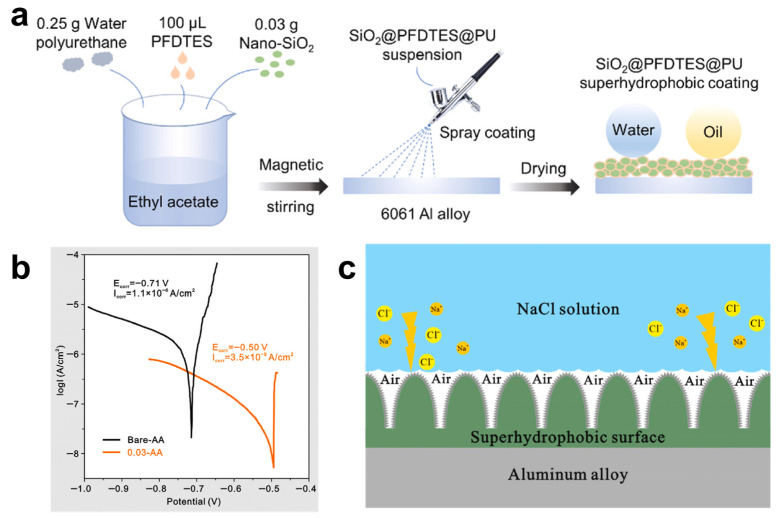
(**a**) Preparation process diagram of SiO_2_@PFDTES@PU coating. (**b**) Potentiodynamic polarization curves of Bare-AA and 0.03-AA sample in 3.5 wt% NaCl aqueous solution. (**c**) Corrosion resistance mechanism diagram of as-prepared superhydrophobic coating [115]. Copyright © 2023 Elsevier Ltd. and Techna Group S.r.l.

One promising strategy to enhance corrosion resistance involves chemical synergy between functional molecules and structured interfaces. For instance, Zhu et al. [114] developed a multifunctional superhydrophobic coating on copper alloy substrates by incorporating perfluorodecylsilane (PFDS), ethylenediaminetetraacetic acid (EDTA), and amorphous calcium oxalate (ACO). EDTA’s chelation capacity suppressed scale deposition, while the stabilized air layer formed by the hierarchical texture improved corrosion resistance, demonstrated by a 3.4-fold increase in electrochemical impedance and a substantial reduction in scaling mass. Similarly, Li et al. [115] applied a nano-SiO_2_-based hybrid coating composed of SiO_2_@PFDTES embedded in a PU matrix onto aluminum alloys. Figure 19 shows the preparation process, corrosion resistance performance, and mechanism of the coating. This composite exhibited a high WCA (168.34°) along with improved charge transfer resistance and reduced corrosion current density, effectively enhancing the substrate’s durability under electrochemical stress.

In addition to chemical passivation, integrated physical and functional features have been employed to address corrosion and environmental stressors simultaneously. Wei et al. [116] designed a fluorine-free superhydrophobic coating for magnesium alloys that combined corrosion inhibition with electrothermal deicing capability. The coating’s hierarchical morphology, constructed in situ, served to minimize electrolyte penetration, while the embedded conductive network enabled active thermal response. This dual-function design reduced corrosion current density and offered rapid anti-icing performance under sub-zero conditions.

Moreover, photocatalytic and biological protection mechanisms offer another frontier for corrosion mitigation. Li et al. [117] fabricated a TiO_2_-containing superhydrophobic coating for aluminum alloys that exhibited both water repellency and photocatalytic activity. This dual-action system not only prevents electrolyte intrusion but also degrades organic contaminants and microbial films, effectively interrupting bio-corrosion pathways and promoting long-term stability in aqueous environments.

To provide a broader context, Zhang and Xu [118] conducted a comparative analysis of superhydrophobic, superamphiphobic, and slippery liquid-infused porous surfaces (SLIPSs). They highlighted how these surfaces create different forms of isolation: superhydrophobic and amphiphobic coatings stabilize trapped air layers, while SLIPSs utilize infused lubricants to form continuous liquid barriers. Both mechanisms effectively reduce ion transport, microbial adhesion, and fouling accumulation, thereby prolonging surface longevity in chemically aggressive environments.

In conclusion, superhydrophobic coatings offer a compelling strategy for corrosion control through passive water repellency, active functional integration, and compatibility with diverse substrates. Their performance can be further optimized by incorporating multifunctional additives (e.g., photocatalysts, binders, conductive phases), tailoring surface architecture, and adopting fluorine-free, bio-based components. Future work should emphasize mechanical durability under abrasion, cyclic environmental exposure, and sustainable scale-up pathways in order to enable practical applications in infrastructure, transportation, and marine engineering.

### 4.4. Anti-Icing

Icing represents a serious operational hazard for aircraft, wind turbines, power grids, and other infrastructures exposed to cold and humid environments. The formation and accumulation of ice not only compromise structural integrity and energy efficiency but also raise critical safety concerns. Superhydrophobic coatings, by significantly reducing water adhesion and delaying ice nucleation, offer an attractive passive solution to mitigate icing. These surfaces mimic natural water-repellent structures, such as lotus leaves and insect wings, by maintaining an air barrier and facilitating rapid droplet rebound before freezing can occur.

**Figure 20:**
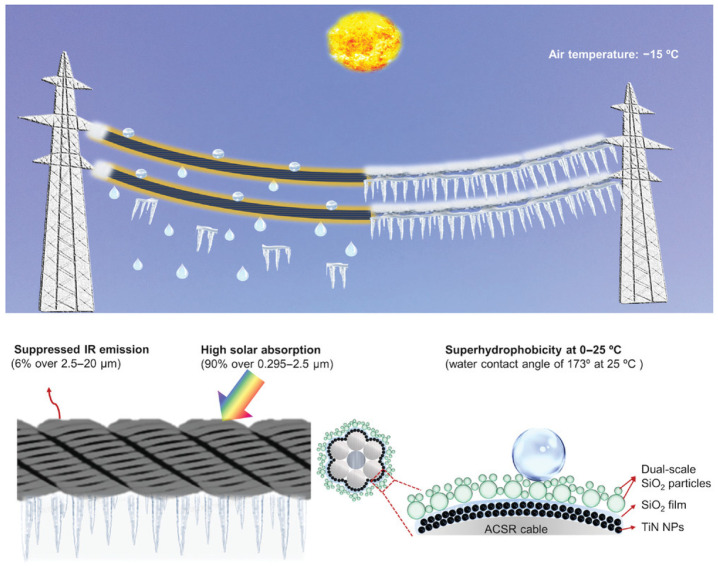
Application of the LE-SS coating on transmission lines and the anti-icing schematic diagram. Reprinted with permission from [120]. Copyright © 2022 Wiley-VCH GmbH.

Recent efforts have explored mechanically robust passive coatings with extended anti-icing functionality. For instance, Wu et al. [119] developed a superhydrophobic surface incorporating Fe_3_O_4_ nanoparticles and a fluorinated resin matrix. This coating endured over 400 tape-peeling cycles while maintaining its performance, demonstrating both durability and delayed ice formation of up to 35 min. The embedded photothermal properties enabled effective infrared-induced de-icing, bridging passive water repellency with an active thermal response. Li et al. [120] developed a low-emissivity solar-assisted superhydrophobic (LE-SS) nanocoating for overhead power lines, combining TiN nanoparticles for solar–thermal conversion and dual-scale SiO_2_ particles for superhydrophobicity. Figure 20 shows a schematic diagram of the anti-icing principle of the LE-SS coating. The coating achieves 90% solar absorptance and 6% IR emissivity, enabling effective deicing/defrosting at −15 °C under 1-sun irradiation by converting solar energy to heat while maintaining superhydrophobicity (WCA of 173° at 25 °C, 145° at 0 °C) to prevent ice adhesion and facilitate ice sliding via hierarchical micro/nanostructures.

In pursuit of hybrid passive–active systems, Zeng et al. [121] designed a superhydrophobic coating integrated with a graphene-based heater for airfoil protection. The hierarchical “honeycomb” and “island” structures delayed icing onset in wind tunnel tests, while the graphene heater provided rapid thermal de-icing. Notably, this combined strategy achieved a 21% reduction in energy consumption compared to heater-only systems, underscoring the potential of dual-function designs for energy-efficient anti-icing.

To prolong anti-icing duration under fluctuating environmental conditions, energy-storage-based approaches have gained attention. Chu et al. [122] reviewed anti-icing strategies using superhydrophobic surfaces, highlighting the limitations of conventional and macrostructured surfaces, and proposed macrostructured photothermal storage superhydrophobic (MPSS) surfaces that integrate photothermal materials and phase change materials (PCMs) to achieve all-day anti-icing via synergistic superhydrophobicity, solar-thermal conversion, and thermal energy storage for applications in aviation, energy, and infrastructure.

Beyond experimental developments, Li et al. [123] evaluated superhydrophobic coatings for power transmission lines, highlighting critical factors such as delayed heterogeneous nucleation, suppressed ice adhesion, and external influences like corona discharge and surface abrasion. Their analysis emphasized the importance of coating longevity and mechanical resilience under field conditions. Similarly, Huang et al. [124] summarized fundamental icephobic mechanisms, including entrapped air layer insulation, reduced solid–liquid heat transfer, and weakened mechanical interlocking. They also noted that cyclic icing/de-icing and wear-induced degradation remain significant hurdles for real-world implementation.

In summary, superhydrophobic coatings present a promising route towards integrated anti-icing solutions that blend passive water repellency with active functionalities such as photothermal conversion and thermal storage. Ongoing research is expected to prioritize material durability under cyclic environmental stress, the enhancement of energy efficiency, and scalable, fluorine-free fabrication routes. These efforts will be essential for translating laboratory breakthroughs into robust technologies for cold-region infrastructure and transportation systems.

### 4.5. Food Applications

Superhydrophobic coatings have been explored increasingly in the food industry due to their combined functionalities of liquid repellency, self-cleaning, and potential antimicrobial activity. These properties address persistent challenges in food packaging and processing, such as residue accumulation, bacterial adhesion, and moisture migration, while also aligning with global trends towards sustainability, food safety, and material biodegradability. A growing body of research demonstrates how naturally derived polymers, edible additives, and fluorine-free fabrication techniques can enable the design of functional surfaces compatible with food-contact environments.

One prominent research direction focuses on biopolymer-based packaging films that integrate water and oil repellency with mechanical robustness and bioactivity. For example, Cui et al. [125] reviewed hydrophobic biopolymer films developed for food packaging, highlighting electrospinning and nanofiller incorporation as effective strategies for increasing surface roughness and reducing surface energy. Many such films also exhibit antibacterial and antioxidant properties, offering dual functionality for extended shelf life and microbial inhibition.

To improve surface hygiene in processing environments, DeFlorio et al. [126] fabricated a fluorine-free superhydrophobic surface on PVC using PDA and nanodiamonds. The resulting structure exhibited a WCA of 151.9° and significantly reduced bacterial adhesion (up to 99%) by maintaining an air cushion in the Cassie–Baxter regime, suggesting its utility for cross-contamination mitigation in food handling facilities.

In addition, natural-fiber-reinforced coatings have demonstrated great promise for biodegradable packaging applications. Frota et al. [127] constructed superhydrophobic coatings by combining silica-functionalized bacterial cellulose nanofibrils with beeswax, achieving a contact angle of 153° alongside excellent thermal stability and self-cleaning ability. Similarly, Lu et al. [128] designed a biodegradable coating based on chitosan and acrylic rosin using a Pickering-emulsion-templating approach. The resultant structure mimicked lotus leaf micro/nano architectures and achieved excellent oil resistance, underscoring its suitability for greaseproof paper packaging.

The development of edible and bioactive films further expands the functionality of superhydrophobic materials in food preservation. Zhang et al. [129] produced a multifunctional edible film composed of chitosan, tea polyphenols, and carnauba wax. This formulation not only delivered superhydrophobic performance (WCA > 100°) but also provided antioxidant capacity, helping to extend the freshness of beef by inhibiting lipid oxidation and moisture absorption.

**Figure 21:**
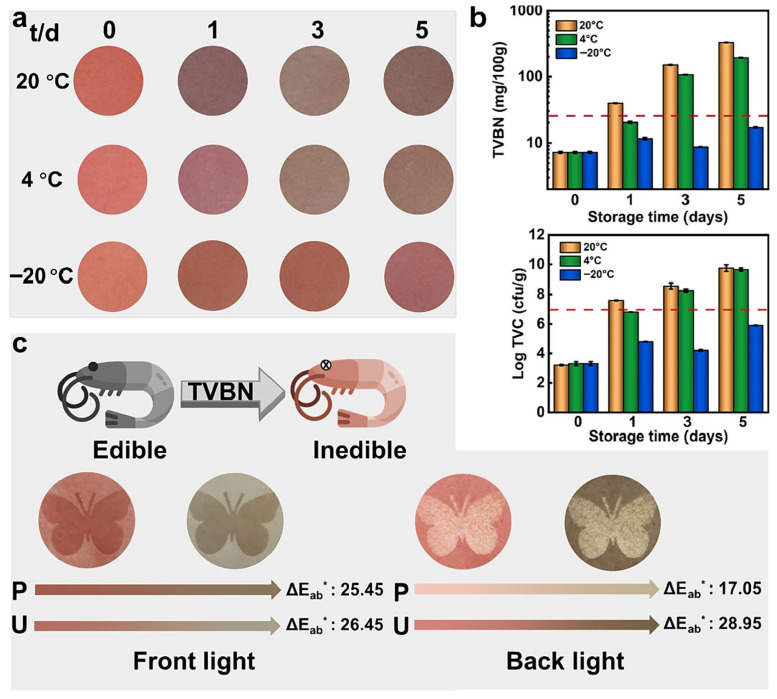
Food freshness monitoring system of PAP-3. (**a**) Monitoring the freshness of shrimp stored at three different temperatures by PAP-3. (**b**) TVBN and TVC values of shrimp at different storage periods and storage temperatures. (**c**) Status of patterned colorimetric label obtained from shrimp stored at 20 °C for 3 days (P: Patterned area, U: Unpatterned area) [130]. Copyright © 2024 Elsevier B.V.

Beyond passive protection, recent advances have explored smart and responsive systems for food quality monitoring. Dong et al. [130] developed a superhydrophobic film with a heteronetwork structure capable of detecting seafood freshness (Figure 21). The film displayed a 126 nm color shift in response to ammonia, enabling real-time spoilage indication. Its hierarchical texture also supported optical anticounterfeiting features, including light-dependent encryption and transparency modulation, demonstrating multifunctionality well beyond simple water repellency.

In applications such as food packaging and biomedical coatings, it is essential to consider the toxicity and environmental behavior of surface modifiers. While silane compounds are effective in lowering surface energy, some of them may present toxicity concerns or leach into food and biological environments. These issues have become increasingly important in recent years as regulations on food-contact materials and biocompatibility become more stringent. To address these concerns, several studies have explored the use of safer alternatives. Natural waxes, polylysine, tannic acid, and food-grade short-chain alkyl silanes have been investigated as promising replacements. For green superhydrophobic coatings used in sensitive contexts, surface modifiers should be selected based on both performance and safety, and their migration potential should be evaluated under realistic use conditions.

In food packaging, the permeability of gases and vapors is a key factor affecting the shelf life of fresh produce such as fruits and vegetables [131,132]. These products rely on a balanced exchange of oxygen and carbon dioxide to sustain respiration during storage. If the permeability of packaging materials is too low, this may lead to anaerobic conditions and accelerate product spoilage. Conversely, if the permeability is too high, this may lead to excessive dehydration and a decline in quality [133,134]. Although superhydrophobic food films are mainly designed for liquid repulsion and barrier properties, factors such as pore size, coating thickness, surface chemistry, and micro/nano roughness can also significantly affect gas exchange kinetics. Future research should focus on the optimization of breathable superhydrophobic coatings that enable moisture control without restricting the natural respiration of fresh foods.

In conclusion, superhydrophobic coatings for food applications offer a versatile platform that combines environmental safety, material sustainability, and intelligent functionality. From biodegradable packaging films and antimicrobial surfaces to freshness sensors and edible coatings, the integration of natural materials and green processing routes continues to drive innovation towards next-generation food contact systems. Future efforts should focus on large-scale fabrication, regulatory compliance, and lifecycle assessment to bridge laboratory concepts with real-world deployment.

### 4.6. Biomedical Applications

Superhydrophobic surfaces have gained increasing attention in biomedical engineering due to their multifunctional advantages, including resistance to bacterial adhesion, reduction of thrombogenicity, and enhanced chemical and mechanical stability under physiological conditions. Inspired by natural antifouling systems, these surfaces combine hierarchical micro/nanostructures with low-surface-energy modifications to create a physical and chemical barrier that mitigates unwanted biological interactions. Their application spans a variety of biomedical contexts, such as implants, blood-contacting devices, wound dressings, and antimicrobial textiles.

One important area of development involves metallic implant surfaces, where superhydrophobicity contributes to corrosion resistance, hemocompatibility, and surface passivation. Weng et al. [135] fabricated a biomimetic superhydrophobic coating on a nitinol alloy using laser cauterization followed by fluorinated alkylsilane (FAS) treatment. The resulting surface exhibited a WCA of 154 ± 1.3° and a significant enhancement in corrosion resistance (up to 2394.1 kΩ·cm^2^). This performance was attributed to the formation of air pockets in the Cassie–Baxter regime, which suppressed electrolyte penetration. Additionally, the coating demonstrated thermal and UV durability, indicating its long-term potential in physiological settings.

Similarly, Chen et al. [136] constructed a superhydrophobic surface on a Ti-6Al-4V biomedical alloy via hydrothermal growth of Na_2_Ti_6_O_13_ nanostructures, followed by PFOTES functionalization. The modified surface achieved a contact angle of 159.2 ± 1.9° and exhibited favorable blood compatibility, as evidenced by reduced hemolysis (2.11%), a prolonged coagulation time, and minimal platelet adhesion. These results highlight the ability of nanostructured hydrophobic surfaces to modulate blood–material interactions through passive bioinertness.

**Figure 22:**
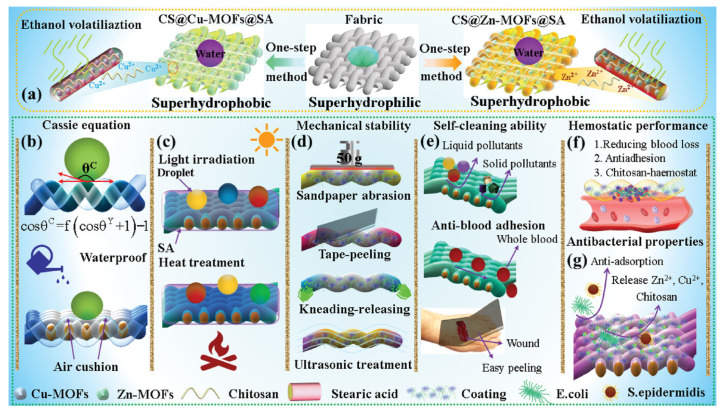
Mechanistic description of (**a**,**b**) water repellent, (**c**) light and thermal stability, (**d**) mechanical stability, (**e**) self-cleaning properties, blood repellency, (**f**) hemostatic properties, and (**g**) antimicrobial properties of superhydrophobic CS@Cu-MOFs@SA and CS@Zn-MOFs@SA composite coatings surface. Reprinted with permission from [137]. Copyright © 2022 Wiley-VCH GmbH.

Beyond rigid implants, superhydrophobic surfaces have also shown promise in soft biomedical interfaces such as wound care materials and antimicrobial textiles. Zhang et al. [137] developed a multifunctional coating on cotton fabric via one-step dip coating using chitosan, stearic acid, and MOF based on Cu or Zn. The coating demonstrated high WCAs (>165°) along with excellent self-cleaning properties. Biomedically, the chitosan–MOF hybrid structure enabled rapid hemostasis (reducing blood loss to 0.004 g) through clot formation, while sustained release of Cu^2+^ or Zn^2+^ ions provided effective antibacterial activity (96.5% inhibition against *E. coli*), making it suitable for advanced wound dressings. Figure 22 shows the application of this multifunctional surface in different fields.

These studies exemplify the multifunctionality of superhydrophobic surfaces in biomedical contexts, where anti-wetting and antifouling performance is often synergistically combined with therapeutic effects such as antimicrobial action or hemostatic efficacy. The key to these outcomes lies in the integration of tailored surface topographies with bioactive or biocompatible modifiers, enabling selective control of material–biology interactions without relying on harsh chemicals.

In conclusion, superhydrophobic coatings offer a versatile and sustainable platform for enhancing the performance of medical materials. Future development should prioritize fluorine-free and biodegradable alternatives, long-term stability under dynamic physiological conditions, and scalable manufacturing techniques in order to meet clinical translation requirements. These coatings represent a promising intersection between materials science, biomedical engineering, and green chemistry.

### 4.7. Other Applications

In addition to the aforementioned domains, superhydrophobic coatings have demonstrated significant versatility in a variety of specialized applications, including biomedical devices, marine engineering, textiles, optics, and self-healing systems. Their multifunctionality, derived from surface micro/nanostructuring and low surface energy materials, has enabled innovations across both industrial and consumer sectors.

In the field of antimicrobial applications, Chen et al. [138] provided a comprehensive review of bioinspired superhydrophobic surfaces, emphasizing how air layers, hierarchical roughness, and low surface energy collaboratively suppress microbial colonization. These features make them promising candidates for use in medical devices and personal protective equipment (PPE) to reduce infection risks. Similarly, Lin et al. [139] fabricated superhydrophobic photothermal coatings using candle soot, combining anti-adhesive and photothermal bactericidal properties to inhibit biofilm formation on biomedical surfaces.

In marine and drag reduction applications, Qu et al. [140] engineered laser-induced superhydrophobic surfaces featuring multiple micro-grooved structures, which achieved substantial drag reduction (up to 66.57% at Reynolds numbers between 2 × 10^5^ and 5 × 10^5^) through sustained air layer entrapment and riblet-like flow modulation. Likewise, Wang et al. [141] employed a one-step laser ablation technique to create bionic fish-scale-inspired superhydrophobic textures, resulting in a 4.814% reduction in hydrodynamic drag via hierarchical structuring and low surface energy.

In textiles and protective materials, Gong et al. [142] developed bioinspired, durable superhydrophobic nanofibrous membranes via humidity-controlled electrospinning and cross-linking. These membranes offered high waterproofness (up to 83.4 kPa) and excellent breathability (3.71 kg·m^−2^·d^−1^), making them suitable for protective clothing. In a similar effort, Zhao et al. [143] created fluorine-free fibrous membranes with high durability, breathability, and self-cleaning properties by engineering nano/microfiber architectures combined with cross-linked coatings, which are ideal for use in next-generation PPE.

In optical and antifogging technologies, Kim et al. [144] designed a multifunctional polymer brush-PFPE sticker (PBPS) grafted onto moth eye nanostructures, achieving excellent anti-reflection (transmittance > 97%) and antifogging performance (>95%) on optical devices such as eyeglasses and goggles. Additionally, Li et al. [145] reviewed biomimetic superwetting surfaces for antifogging, comparing superhydrophobic strategies that trap air to repel water and superhydrophilic strategies that promote uniform filmwise condensation, with applications in lenses, endoscopes, and other optical instruments.

In recent years, the integration of superhydrophobic materials into flexible and wearable electronics has attracted growing attention. These applications demand coatings that are not only water-repellent but also stretchable, breathable, and mechanically robust. Superhydrophobic films deposited on elastic substrates such as PDMS, polyurethane, or conductive textiles have been employed to protect circuits from moisture damage, minimize signal drift due to sweat or rain, and to maintain performance under repeated bending and deformation. Gao et al. [146] developed a two-layer conductive superhydrophobic coating on cotton fabric using silver nanoparticles (inner conductive layer) and modified SiO_2_/waterborne polyurethane (outer superhydrophobic layer, WCA of 158.9°), enabling its use in flexible wearable electronics for underwater motion monitoring and safety alerts. Sneha et al. [147] fabricated highly conductive Au fabric electrodes via LbL assembly, then coated them with PDMS to achieve superhydrophobicity (WCA of 140°), which enhanced stability in wearable supercapacitors with high areal capacitance (660 mF/cm^2^) and durability under mechanical deformations. Suryaprabha et al. [148] prepared superhydrophobic cotton fabrics by coating MWCNT/ferrite/Ni chains followed by PDMS (WCA of 156°), exhibiting self-cleaning, electromagnetic-shielding (~54 dB), and underwater-Morse-code-signaling capabilities, suitable for multifunctional wearable electronics.

These diverse applications underscore the broad impact and adaptability of superhydrophobic materials across multiple disciplines. Their continued optimization will be critical for advancing multifunctional surface technologies in both emerging and established fields. The available industrial examples underscore the practical relevance and emerging adoption of superhydrophobic technologies. For instance, NTT-AT (Tokyo, Japan) has commercialized a water-based superhydrophobic coating, HIREC^®^ 300-W, which achieves a WCA over 150° and maintains self-cleaning performance outdoors for around 3 years with minimal VOC content. This product demonstrates that sustainable, fluorine-free coatings can meet long-term durability and environmental safety standards relevant to industry. Meanwhile, HeiQ (Schlieren, Switzerland) has introduced the Eco Dry textile technology, a fluorocarbon-free, highly durable water-repellent finish that mimics a duck’s feather structure and has been adopted by major outdoor apparel brands such as The North Face and Jack Wolfskin. These real-world implementations reinforce the feasibility of green superhydrophobic solutions at a commercial scale and exemplify the trend toward environmentally compliant coatings.

## 5. Challenges and Development Trends

### 5.1. Current Challenges

Despite remarkable advancements, several critical challenges hinder the large-scale deployment and commercialization of green superhydrophobic coatings.

Durability remains a key issue. Many biopolymer-based coatings (e.g., cellulose, starch) exhibit mechanical fragility or instability in humid environments. Achieving long-term durability, such as abrasion resistance or weathering tolerance, without compromising biodegradability, is complex. While reinforcing bio-based coatings with nanoparticles or introducing synthetic additives can enhance robustness, such modifications often diminish environmental compatibility. Although the material selection and sustainable fabrication strategies for green superhydrophobic surfaces were emphasized, it should be noted that mechanical durability and environmental stability are equally critical for practical deployment. Key aspects such as abrasion resistance, chemical stability under acidic or alkaline conditions, UV resistance, and thermal cycling remain insufficiently addressed in the context of green coatings. In addition to chemical resistance and long-term environmental stability, practical applications require superhydrophobic coatings to retain their performance under repeated use. This includes exposure to mechanical stresses such as bending, stretching, abrasion, and laundering, particularly for flexible substrates like textiles or wearable electronics. However, systematic studies that quantify the loss of superhydrophobicity over cycles of deformation or washing remain limited, especially in the context of biodegradable or green coatings. Future research should thus prioritize the development of standardized, repeatable durability assessments, including washing cycles, cyclic compression, and fatigue testing, to guide material optimization and ensure reliability across real-world applications.

Adhesion and surface integration are also crucial. Green coatings must bond firmly to diverse substrates, including metals, glass, wood, and textiles. However, natural polymers often lack intrinsic adhesion. To address this, researchers are exploring benign primers, natural binders (e.g., tannic-acid-based adhesives), and surface activation techniques such as plasma treatment or mild laser ablation. Still, scalable, eco-friendly, and substrate-universal application methods remain under development. Substrate-specific adhesion strategies have also been explored in recent years to address this issue in practical applications. For glass substrates, which are smooth and rich in surface hydroxyl groups, silicon-based coatings have shown excellent compatibility. For instance, Huang et al. [149] prepared a superhydrophobic coating on binder-pre-treated glass by spraying, which had a strong adhesion (withstanding 50 wears of 240 grains of sandpaper under a 100 g load), and the WCA was >150°. Research by Chen et al. [36] also confirmed that the glass surface is smooth and rich in hydroxyl groups, and has good compatibility with silicon-based green coatings. For metal substrates, enhanced interfacial adhesion is crucial for preventing delamination under mechanical or corrosive stress. Yuan et al. [150] formed a porous Al_2_O_3_ framework through cathode plasma electrolytic deposition, then electrophoretically deposited PTFE into the pores. The Al_2_O_3_ layer ensures strong mechanical adhesion to the metal substrate, enabling the coating to withstand 50 wear cycles of 240 grains of sandpaper under a 100 g load, while PTFE endows it with superhydrophobicity (WCA 162.7°). Yue et al. [151] enhanced the adhesion of the coating to low-carbon steel through layering assembly, endowing the metal substrate with long-term corrosion resistance, with a WCA of 150°. For polymeric substrates, the challenges lie in low surface energy and limited reactive groups. Xia et al. [152] employed interfacial strengthening cells derived from natural diatomite to improve chemical compatibility with epoxy coatings. Wang et al. [153] developed coatings from recycled polypropylene and PDMS/nano-SiO_2_ composites that maintained hydrophobicity (WCA > 130°) after 100 abrasion cycles, demonstrating potential in plastic recycling. These examples collectively highlight the importance of substrate-specific interfacial engineering in the practical deployment of green superhydrophobic technologies.

Solvent use and processing conditions pose further limitations. Although many systems are waterborne, some still rely on organic solvents or require energy-intensive curing (e.g., UV or thermal). Future commercialization demands ambient-condition fabrication techniques with minimal volatile organic compounds, ideally using aqueous-phase chemistries and energy-efficient methods [154,155].

### 5.2. Development Trends

To overcome these limitations, current research is trending toward multifunctional and sustainable material systems. Innovative coatings now integrate self-healing, antimicrobial, anti-icing, and flame-retardant capabilities without sacrificing environmental compatibility. For instance, lignin imparts UV resistance, while chitosan provides inherent antibacterial activity. Microencapsulation and hybrid nanocomposites (e.g., clay–biopolymer matrices) are being adopted to improve mechanical strength and functional responsiveness.

Scalable and benign fabrication techniques are a key focus. Water-based sol–gel methods, spray- or dip-coating processes, and electrospinning of biopolymers are being optimized for industrial use. Concurrently, attention is shifting toward fully biodegradable architectures, including coatings designed to degrade harmlessly after their functional life, such as sacrificial fibers or transient adhesives. However, research gaps persist in areas such as the integration of superhydrophobic materials with advanced manufacturing techniques like three-dimensional printing, the toxicological evaluation of surface modifiers in biological and food-related applications, and the lack of quantitative cost analyses for large-scale production. Standardized performance testing protocols and life-cycle assessments are also needed to ensure both safety and sustainability. Future research efforts are therefore encouraged to incorporate these dimensions into material design and engineering strategies, helping to bridge the gap between laboratory-scale developments and industrial-scale adoption.

Predictive modeling and design are gaining traction, especially for understanding the relationships among surface roughness, surface energy, and liquid repellency in natural polymers. Moreover, emerging frontiers include novel biomass feedstocks (e.g., algal chitosan, fungal mycelium) and even engineered living coatings, such as microbial films designed for water repellency or self-repair. Moreover, encouraging regulatory shifts, particularly restrictions on per- and polyfluoroalkyl substances, are driving both academic and industrial communities towards safer, greener alternatives. The convergence of nanotechnology, polymer science, and environmental engineering is expected to yield next-generation green coatings with programmable surface properties and broad applicability.

### 5.3. Economic Considerations and Industrial Feasibility

Material cost and resource availability represent practical constraints. While bio-based materials like nanocellulose or chitosan may incur high production costs, the use of agricultural or marine waste biomass (e.g., bacterial cellulose, algae-derived chitosan) offers potential solutions. Maintaining quality and performance parity with inexpensive fluoropolymers remains a substantial challenge, however. Although many green superhydrophobic coatings have demonstrated promising performance in laboratory-scale studies, their transition to industrial-scale production remains constrained by cost-related factors. The economic feasibility of these technologies depends not only on raw material cost and availability, but also on the energy requirements, equipment complexity, throughput, and compatibility with existing manufacturing infrastructure.

Among the reviewed fabrication methods, spray coating and dip coating are generally considered the most economically viable for scale-up due to their operational simplicity, low energy input, and adaptability to roll-to-roll processes. These techniques are particularly suited for coating flexible substrates such as textiles, packaging films, and large-area panels. Sol–gel processes also offer scalability, especially when using water- or ethanol-based systems, although the cost of silica precursors and drying time may pose limitations. On the other hand, methods such as electrospinning and laser ablation, while effective for producing highly controlled micro/nanostructures, are currently limited by high capital investment, slow processing speed, and batch-mode operation, which restrict their cost-efficiency for high-volume manufacturing. Self-assembly techniques, although potentially scalable, often suffer from low throughput and long processing times.

Moreover, beyond production costs and scale-up feasibility, consideration of the coating’s end-of-life behavior is essential for a truly sustainable application. Most durable coatings are formulated for strong adhesion or cross-linked networks, making removal challenging without damaging the substrate. Such removal processes often require aggressive mechanical or solvent-based treatments that may degrade underlying materials, increasing waste and economic burden. As an alternative, emerging research has focused on recyclable or degradable superhydrophobic systems. One recent study introduced a reproducible coating that offered both high wear resistance and recyclability using thermoplastic polyurethane and silica nanoparticle composites, enabling anti-icing properties along with substrate reuse [156]. These design strategies not only align with green chemistry principles but also reduce disposal costs and support a circular-use model for coated materials. Future techno-economic analyses should encompass not only production and performance but also the removability, recovery, and reuse of coatings to fully represent life-cycle sustainability.

Overall, while green superhydrophobic materials present substantial promise for sustainable innovation, further research is required to develop cost-effective, scalable, and durable systems that meet industrial standards. Techno-economic analyses and pilot-scale demonstrations will be the critical next steps to evaluate real-world feasibility.

## 6. Conclusions

Green superhydrophobic coatings have evolved from a niche concept to a rapidly advancing interdisciplinary research frontier. By utilizing natural polymers such as cellulose, lignin, chitosan, starch, and silk fibroin, combined with sustainable modification strategies, researchers have developed surface coatings that simultaneously possess water repellency, environmental compatibility, and functional versatility. These materials represent a fundamental shift from fluorinated and petroleum-derived compounds towards safer, biodegradable, and more sustainable alternatives.

This review systematically discussed the material foundations, fabrication strategies, and application domains of green superhydrophobic surfaces. We categorized natural substrates by source and properties, reviewed scalable and eco-friendly fabrication techniques including sol–gel, dip coating, electrospinning, and laser texturing, and highlighted their performance in applications such as self-cleaning, corrosion resistance, anti-icing, oil–water separation, and biomedical use. Importantly, we also discussed challenges related to mechanical durability, long-term stability, and commercial scalability, offering insight into scientific and practical limitations that must be overcome.

In the future, the integration of smart responsive functionalities (e.g., sensing, self-healing), fluorine-free surface chemistry, and cost-effective large-area processing will be critical in achieving widespread industrial adoption. Cross-disciplinary collaboration between material science, chemical engineering, and environmental science will be essential in propelling this technology from lab-scale demonstration to commercial reality.

## Figures and Tables

**Table 1:** Comparison of representative natural materials for green superhydrophobic coatings.

Material	Source Type	Tensile Strength (MPa)	Processability	Approx. Cost Level	Biodegradability	Green Modification Potential	References
Cellulose	Plant (wood, cotton)	200–1000 (nanofibers)	Films, fibers, aerogels	Low	Fully biodegradable	High	[36,65,66]
Chitosan	Animal (crustaceans)	30–70	Films, hydrogels	Medium	Fully biodegradable	High	[33,48,67]
Lignin	Plant (wood waste)	5–80 (varies by type)	Fillers, blends	Very low	Partially biodegradable	Moderate	[44,68,69]
Starch	Plant (corn, potato)	2–40	Films, foams, blends	Very low	Fully biodegradable	Moderate–High	[22,50,70,71]
Silk Fibroin	Animal (silkworm cocoons)	300–740	Fibers, films, sponges	High	Fully biodegradable	High	[55,56,72,73]

## Data Availability

No new data were created or analyzed in this study. Data sharing does not apply to this article.

## Abbreviations

The following abbreviations are used in this manuscript:

SA	Sliding Angle
CNCs	Cellulose Nanocrystals
NFC	Nanofibrillated Cellulose
CVD	Chemical Vapor Deposition
WCA	Water Contact Angle
AAO	Anodized Aluminum Oxide
OTS	Octadecyltrichlorosilane
PFDTES	Perfluorodecyltriethoxysilane
HT-LNS	Heat-Treated Lignin Nanospheres
PU	Polyurethane
PDMS	Polydimethylsiloxane
PAni–ZnO–STA	Chitosan–Polyaniline–Zno–Stearic Acid
PTA–CS	Chitosan–Tannic Acid Complex
SPC	Starch, Polyhydroxyurethane, and Cellulose Nanocrystals
HPMC	Hydroxypropyl Methylcellulose
SNPs	Starch Nanoparticles
SFP	Silk Fibroin Powder
SPI	Soybean Protein Isolate
CGTS	Chitosan/Gelatin/SiO_2_
HS–SH–F	Halloysite Nanotube–Silica Hybrid Particles
IPN	Interpenetrating Polymer Network
SHPM	Superhydrophobic Copper Mesh
PVDF	Polyvinylidene Fluoride
PDA	Polydopamine
RGO	Reduced Graphene Oxide
PFOTES	Perfluorooctyltriethoxysilane
HDTMS	Hexadecyltrimethoxysilane
TEOS	Tetraethoxysilane
MTES	Methyltriethoxysilane
TMCS	Trimethylchlorosilane
LbL	Layer-by-Layer
PTFE	Polytetrafluoroethylene
SEBS	Styrene–Ethylene/Butylene–Styrene
SRCF	Radiative Cooling Fabrics
MWCNTs	Multi-Walled Carbon Nanotubes
PEI	Polyethyleneimine
TMSPA	Trimethoxysilane Propyl Acrylate
DTMS	Dodecyltrimethoxysilane
PFDS	Perfluorodecylsilane
EDTA	Ethylenediaminetetraacetic Acid
ACO	Amorphous Calcium Oxalate
SLIPS	Slippery Liquid-Infused Porous Surfaces
LE-SS	Low-Emissivity Solar-Assisted Superhydrophobic
MPSS	Macrostructured Photothermal Storage Superhydrophobic
PCMs	Phase Change Materials
FAS	Fluorinated Alkylsilane
PPE	Personal Protective Equipment
PBPS	Polymer Brush-PFPE Sticker
